# PSKH1 kinase activity is differentially modulated via allosteric binding of Ca^2+^ sensor proteins

**DOI:** 10.1073/pnas.2420961122

**Published:** 2025-02-18

**Authors:** Christopher R. Horne, Toby A. Dite, Samuel N. Young, Lucy J. Mather, Laura F. Dagley, Jared L. Johnson, Tomer M. Yaron-Barir, Emily M. Huntsman, Leonard A. Daly, Dominic P. Byrne, Antonia L. Cadell, Boaz H. Ng, Jumana Yousef, Dylan H. Multari, Lianju Shen, Luke M. McAloon, Gerard Manning, Mark A. Febbraio, Anthony R. Means, Lewis C. Cantley, Maria C. Tanzer, David R. Croucher, Claire E. Eyers, Patrick A. Eyers, John W. Scott, James M. Murphy

**Affiliations:** ^a^Walter and Eliza Hall Institute of Medical Research, Parkville, VIC 3052, Australia; ^b^Department of Medical Biology, University of Melbourne, Parkville, VIC 3052, Australia; ^c^Drug Discovery Biology, Monash Institute of Pharmaceutical Sciences, Monash University, Parkville, VIC 3052, Australia; ^d^Meyer Cancer Center, Weill Cornell Medicine, New York, NY 10021; ^e^Department of Cell Biology, Harvard Medical School, Boston, MA 02115; ^f^Dana-Farber Cancer Institute, Harvard Medical School, Boston, MA 02215; ^g^Englander Institute for Precision Medicine, Institute for Computational Biomedicine, Weill Cornell Medicine, New York, NY 10021; ^h^Columbia University Vagelos College of Physicians and Surgeons, New York, NY 10032; ^i^Department of Biochemistry, Cell and Systems Biology, Institute of Systems, Molecular and Integrative Biology, University of Liverpool, Liverpool L69 7ZB, United Kingdom; ^j^Centre for Proteome Research, Institute of Systems, Molecular and Integrative Biology, University of Liverpool, Liverpool L69 7ZB, United Kingdom; ^k^Cancer Ecosystems Program, Garvan Institute of Medical Research, Sydney, NSW 2010, Australia; ^l^School of Behavioural and Health Sciences, Australian Catholic University, Fitzroy, VIC 3065, Australia; ^m^St. Vincent’s Institute of Medical Research, Fitzroy, VIC 3065, Australia; ^n^NuaBio Research, Burlingame, CA 94010; ^o^Molecular and Cellular Biology, Baylor College of Medicine, Houston, TX 77030; ^p^School of Clinical Medicine, Faculty of Medicine and Health, University of New South Wales Sydney, Sydney, NSW 2010, Australia; ^q^The Florey Institute of Neuroscience and Mental Health, Parkville, VIC 3052, Australia

**Keywords:** protein kinase, calmodulin, UNC119B, reticulocalbin, allostery

## Abstract

Protein Serine Kinase H1 (PSKH1) was recently identified as a crucial factor in kidney development and is overexpressed in prostate, lung, and kidney cancers. However, little is known about PSKH1 regulatory mechanisms, leading to its classification as a “dark” kinase. Here, we used biochemistry and mass spectrometry to define PSKH1’s consensus substrate motif, protein interactors, and how interactors, including Ca^2+^ sensor proteins, promote or suppress activity. Intriguingly, despite the absence of a canonical Calmodulin binding motif, Ca^2+^-Calmodulin activated PSKH1 while, in contrast, the ER-resident Ca^2+^ sensor of the Cab45, Reticulocalbin, Erc55, Calumenin (CREC) family, Reticulocalbin-3, suppressed PSKH1 catalytic activity. In addition to antagonistic regulation of the PSKH1 kinase domain by Ca^2+^ sensing proteins, we identified UNC119B as a protein interactor that activates PSKH1 via direct engagement of the kinase domain. Our findings identify complementary allosteric mechanisms by which regulatory proteins tune PSKH1’s catalytic activity and raise the possibility that different Ca^2+^ sensors may act more broadly to tune kinase activities by detecting and decoding extremes of intracellular Ca^2+^ concentrations.

## Significance

The approximately one-third of human kinases that are considered understudied or “dark” kinases offer a treasure trove of possibility for uncovering new mechanisms of kinase regulation. Here, we describe how protein interactors can activate or suppress catalysis by the dark kinase, Protein Serine Kinase H1 (PSKH1). By direct binding to the kinase domain, the Ca^2+^ sensor protein, Calmodulin, elevates PSKH1 activity, while members of the Golgi-resident CREC family inhibit activity. An unrelated interactor, UNC119B, best characterized as an acyl chain binder, was also identified to act as an allosteric activator of the PSKH1 kinase domain. These studies expand the repertoire of mechanisms employed in kinase regulation and rationalize how extremes in Ca^2+^ flux can be decoded to tune kinase activities.

The human kinome was documented more than 20 y ago, yet the attention of the research community has focused on a small subset of kinases that have been disproportionately subjected to detailed study and targeting by small molecule inhibitors. In contrast, approximately one-third of the human kinome has been classified as understudied “dark” kinases (1), with 22 of these 160 dark kinases residing in the Ca^2+^-Calmodulin-dependent kinase (CAMK) family. Interest in the biological functions of one such CAMK member, Protein Serine Kinase H1 (PSKH1), has recently grown owing to its implication as a crucial factor in kidney development and the association of PSKH1 overexpression with several cancers, including those of the prostate, lung, and kidney (2). Recently, three substitutions in the PSKH1 kinase domain that were identified in patients with kidney ciliopathies were found to suppress PSKH1 catalytic activity (3), providing a direct link between PSKH1 kinase activity and kidney development.

Among the few studies to date, PSKH1 was reported to reside in the Golgi owing to respective myristoylation and palmitoylation on the conserved residues, Gly2 on Cys3 (4). The precise roles of these posttranslational modifications on PSKH1 catalytic activity, however, are not known. Equally, knowledge of PSKH1 interactors and substrates is scant, warranting detailed examination of its interactome and the influence of binders on catalytic regulation. Recent data implicate the Ca^2+^ sensor protein, Calmodulin, as an inhibitor of the activity of another CAMK family member, CHK2 (5), and Calmodulin had been previously reported to inhibit PSKH1 activity (6). Like PSKH1, CHK2 lacks a conventional Calmodulin binding motif, raising the prospect that PSKH1 may similarly be regulated by Ca^2+^ sensor proteins via an unconventional binding mode. Calmodulin is a ubiquitous intracellular protein in eukaryotic cells, which constitutes up to 0.1% of the total proteome in overall abundance and serves a crucial function as a Ca^2+^ sensor (7). The cellular influx of Ca^2+^ is a critical second messenger in many facets of biology, from muscle contractility to neuronal communication (7, 8). Calmodulin binding to four Ca^2+^ ions promotes a conformational change that enables engagement of downstream signaling effectors to regulate their activities through a range of mechanisms, including localization within cells, the occlusion of substrate or partner protein binding, or by modulating a binding partner’s catalytic activity.

Calmodulin is a bilobed, four EF-hand fold protein, which typically binds 15 to 30 amino acid Calmodulin binding motifs in target proteins with up to 20 nM affinity (9, 10). Our recent data expand the repertoire of Calmodulin binding modes to include regulation of a kinase by direct binding to the kinase domain via a 3D interface on the surface (5), rather than by binding to a conventional linear sequence motif. Whether Calmodulin regulates PSKH1 activity via a similar mode is yet to be examined. Additionally, PSKH1’s Golgi localization raises the prospect that further Ca^2+^ sensor proteins in the secretory pathway might contribute to regulation of its catalytic activity (6). The Cab45, Reticulocalbin, Erc55, Calumenin (CREC) family of Ca^2+^ sensors is known to operate, at least in part, at the Golgi and endoplasmic reticulum (11), although members are poorly characterized and, unlike Calmodulin, are yet to be implicated in the regulation of kinase activity. Calmodulin is known to bind Ca^2+^ with micromolar affinity to undergo a conformation change that tunes its binding repertoire (10), and in the presence of a target enzyme, such as MLCK, Calmodulin affinity for Ca^2+^ is vastly elevated to nanomolar range (7, 12). In contrast, biochemical studies indicate that CREC family sensors undergo an unstructured to structure transition at millimolar Ca^2+^ concentrations (13, 14), raising the prospect that Calmodulin and CREC proteins may serve complementary roles in Ca^2+^ sensing and kinase regulation in cells. The impact of Calmodulin and CREC proteins on PSKH1 activity therefore is of considerable interest.

Here, we use biochemical and mass spectrometry approaches to define the mechanism of PSKH1 regulation. We identify antagonistic roles for two types of Ca^2+^ sensor, Calmodulin and members of the CREC family, in modulating PSKH1 catalytic activity, via direct binding to the PSKH1 kinase domain. In contrast to the Calmodulin-mediated inhibition of catalytic activity recently observed for CHK2 (5), Calmodulin binding to the PSKH1 catalytic domain promoted enzymatic activity. Another protein identified as proximal to PSKH1 by mass spectrometry, UNC119B, attracted our interest because of its reported contribution to cilia formation (15, 16), as recently defined for PSKH1 (5). UNC119B is best known as an acyl chain binder (17), through which activation of Src family kinases and Ras family GTPases has previously been described (18). Unexpectedly, however, mutation or truncation of the N-terminal myristoylation and palmitoylation sites in PSKH1 did not dampen UNC119B-mediated PSKH1 activation, identifying direct binding to the PSKH1 kinase domain as another mechanism by which UNC119B can promote kinase activity. Our findings illustrate how coordinated interactions with allosteric regulators can complementarily operate to tune kinase catalytic activity. In particular, our data suggest a rheostatic mechanism by which low Ca^2+^ could license Calmodulin to activate kinase activity, while high Ca^2+^ concentrations enable CREC family Ca^2+^ sensors to dampen catalytic activity, to decode extremes in Ca^2+^ flux.

## Results

### PSKH1 Substrate Consensus Motif.

**Fig. 1.**
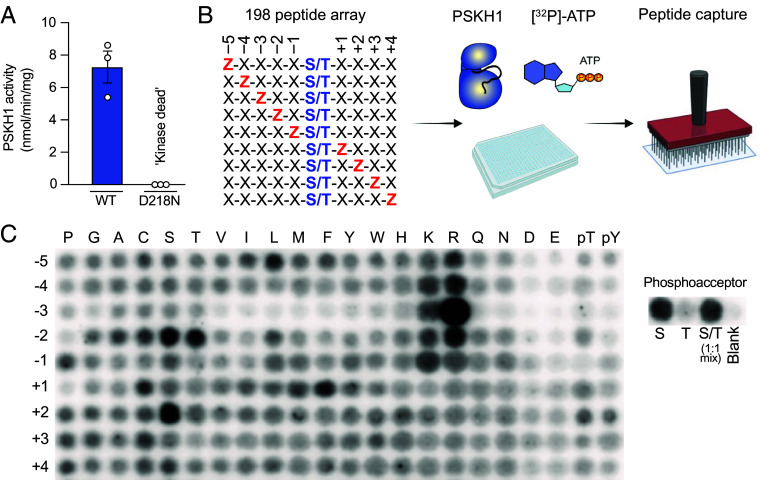
PSKH1 is a serine kinase that favors basic residues at the -3 position. (*A*) Radiometric assay of recombinant full-length PSKH1 expressed in insect cells. Wild-type, but not D218N kinase-dead, recombinant PSKH1 robustly phosphorylates the ADR1 peptide. (*B*) Schematic of experimental workflow for positional scanning peptide analysis (PSPA) and representative results; schematic created using BioRender. Z denotes fixed positions containing one of the 20 natural amino acids, or either phosphorylated Thr (pT) or phosphorylated Tyr (pY). X denotes variable positions containing randomized mixtures of all natural amino acids except Ser, Thr, and Cys. (*C*) Radiometric PSKH1 kinase assay performed on peptide array, where darker spots indicate preferred residues. S, G-A-X-X-X-X-X-S-X-X-X-X-A-G-K-K(LC-biotin); T, G-A-X-X-X-X-X-T-X-X-X-X-A-G-K-K(LC-biotin); where X = degenerate mixture of the 16 natural amino acids excluding cysteine, tyrosine, serine, and threonine. All data in panel *C* and *SI Appendix*, Fig. S1 are extracted from the same peptide array experiment.

As with many understudied kinases, knowledge of PSKH’s physiological substrates and site specificity is scant. To define a consensus substrate motif, we first established methods for robust expression and purification of recombinant PSKH1 from insect cells. Initially, we trialed recombinant wild-type PSKH1 in a radiometric kinase assay with peptide substrates known to be phosphorylated by other CAMK family kinases and identified the CAMK4 substrate, ADR1, as a target for PSKH1 (Fig. 1*A*). We confirmed this activity was attributable to PSKH1 and not a contaminating kinase by comparing the activities of wild-type and D218N kinase-dead mutant PSKH1, with the latter incapable of measurable phosphotransfer (Fig. 1*A*).

Subsequently, we employed positional scanning peptide array analysis (19–22) as an unbiased approach to define the consensus substrate-recognition motif of PSKH1. This method uses an arrayed combinatorial peptide library in which each of nine positions surrounding a central Ser or Thr phosphoacceptor residue is systematically substituted to each of the 20 natural amino acids in addition to phospho-Thr or phospho-Tyr (Fig. 1*B*). This approach was recently used to define the substrate preferences of 303 human Ser/Thr kinases (21), although not all Ser/Thr kinases were able to be profiled in this study, including PSKH1. PSKH1 showed a strong preference for Ser over Thr as the phosphoacceptor residue. This preference is consistent with Leucine occurring as the DFG+1 residue in the PSKH1 activation loop: a hallmark of selectivity toward Serine substrates in kinase families except the CMGC kinases (23). Like many CAMK group kinases (21), PSKH1 prefers basic residues N-terminal to the phosphoacceptor residue, with a clear preference for Arg at the -3 position. While Arg is preferred at -3, Lys, but not acetylated or trimethylated Lys, was tolerated (*SI Appendix*, Fig. S1), emphasizing the required basicity of the -3 residue for PSKH1 recognition. Additionally, while most residues are tolerated at the +1 site, there is an overt preference for Phe, leading to the consensus substrate motif: L/R-X-R-T/R-X-(S)-F-X-X-X where (S) is the phosphoacceptor and X is any amino acid (Fig. 1*C*). Serendipitously, the ADR1 peptide sequence, LKKLTRRA(S)FSGG, conforms to the experimentally derived PSKH1 substrate consensus sequence, which underscores the suitability of ADR1 as a peptide substrate in our radiometric assays. We also noted that the position of the phosphoacceptor could be varied in 7mer peptides, with all but the C-terminal position tolerated, whereas 4mer peptides were poor substrates (*SI Appendix*, Fig. S1).

### PSKH1 Binds Secretory Network Ca^2+^ Sensor Proteins and the Adaptor, UNC119B.

**Fig. 2.**
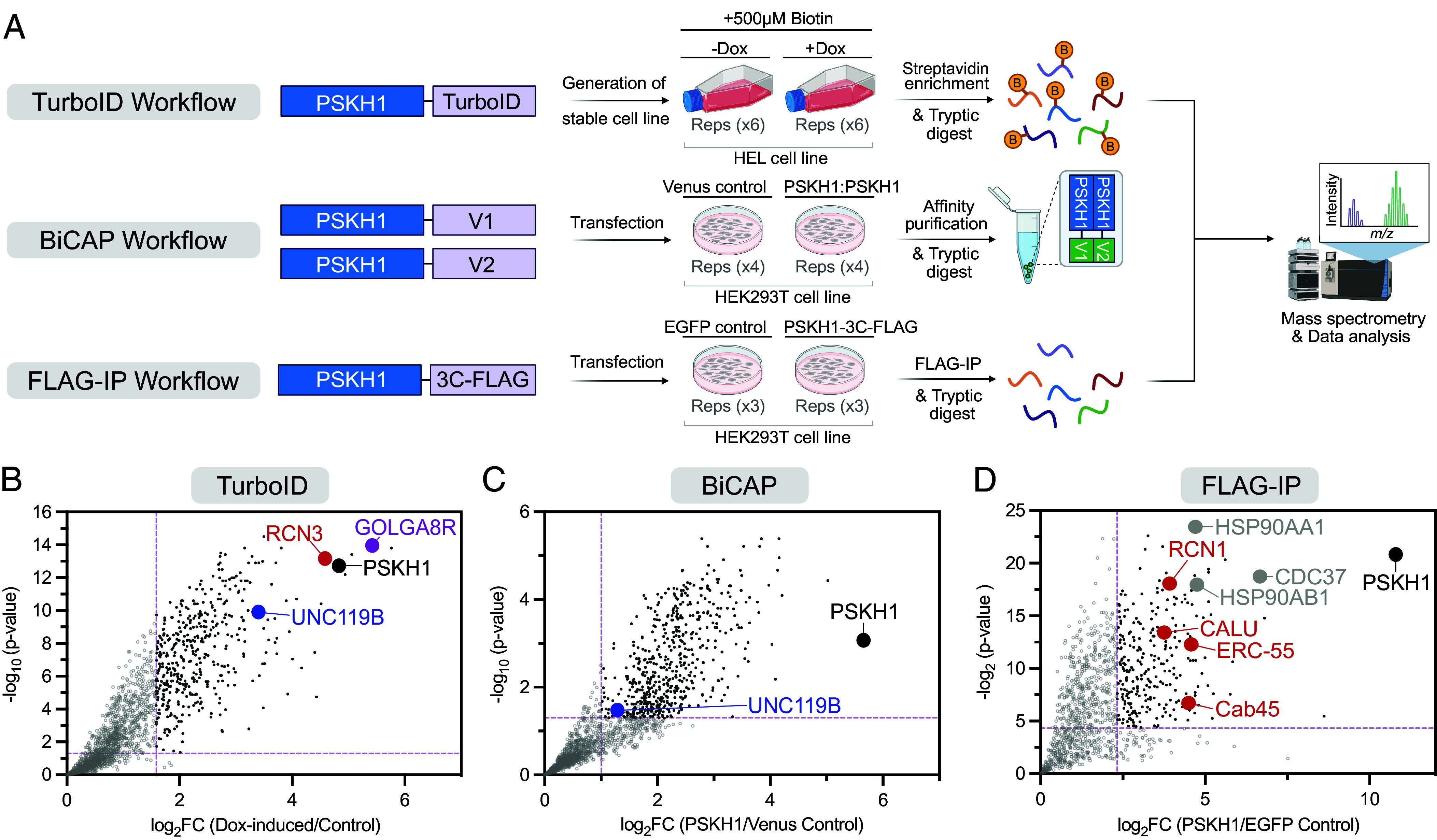
Mapping the PSKH1 interactome using complementary proteomic approaches. (*A*) Schematic of each experimental workflow; created using BioRender. (*B*) Volcano plot of TurboID proximity labeling experiment in HEL cells with PSKH1 and proximal proteins of interest highlighted. Pink dashed lines denote a threefold change and *P*-value < 0.05. Data are representative of six independent experiments. (*C*) Volcano plot of BiCAP, validating interaction of dimerized PSKH1 with UNC119B in HEK293T cells. Pink dashed lines denote a twofold change and *P*-value < 0.05. Data are representative of four independent experiments. (*D*) Volcano plot of FLAG-IP, validating interaction of PSKH1 with CREC family members (red dots) and the Cdc37-HSP90 chaperone system (gray). Pink dashed lines denote a fivefold change and *P*-value <0.05. Data are representative of three independent experiments. In all panels, PSKH1 is highlighted in black, UNC119B in blue, Calcium sensing interactors of the CREC family in red, secretory pathway interactors in purple, and chaperone interactors in gray. Significantly enriched hits are depicted as closed black circles and nonsignificant hits are depicted as open gray circles.

We next sought to identify proteins that bind to, and potentially regulate the activity of, PSKH1 using complementary proteomics approaches (Fig. 2*A*). Initially, we C-terminally tagged PSKH1 with the TurboID variant of BirA (24), to facilitate rapid promiscuous biotinylation of proteins proximal to PSKH1 upon induction of its expression with doxycycline in HEL cells. HEL cells are a human erythroleukemia line that was selected for TurboID experiments because of their endogenous expression of PSKH1 and the paralogous pseudokinase, PSKH2 (25). PSKH2 lacks the critical Asp of the catalytic loop HRD motif that is required for kinase activity and, while this substitution ablates catalytic activity, PSKH2’s cellular function remains to be determined (26). Relative to uninduced cells, PSKH1 and secretory network proteins, including GOLGA8R, a protein resident within the Golgi, Reticulocalbin-3 (RCN3) of the CREC family, and the adaptor protein, UNC119B, were most enriched following Streptavidin affinity precipitation, tryptic digest and mass spectrometry analysis (Fig. 2*B*). Consistent with an absence of reports of PSKH1:PSKH2 interaction in interactome databases, such as BioGRID, PSKH2 was not identified in the PSKH1 proximitome in HEL cells, suggesting that PSKH1 and PSKH2 do not heterodimerize under basal conditions. The absence of PSKH2 in the PSKH1 TurboID proximitome was not a consequence of PSKH2 expression lacking in HEL cells, because PSKH2 could be detected in immunoblots of HEL cell lysates using a monoclonal antibody that we raised against a peptide unique to human PSKH2 (*SI Appendix*, Fig. S2*A*). These data assert that under basal conditions, formation of a PSKH1:PSKH2 heterocomplex does not occur, although we recognize that there may be cellular contexts under which this interaction occurs that remain to be identified.

Because PSKH1 is a CAMK family kinase, we further investigated whether increased Ca^2+^ influx can modulate these interactions by performing parallel TurboID experiments in the presence of ionomycin to induce Ca^2+^ influx. These data reveal no major changes in the proximitome of PSKH1 upon increased Ca^2+^ flux (*SI Appendix*, Fig. S2*B*), nor any major changes in gene ontology (GO) analyses of significantly enriched hits in the presence and absence of ionomycin treatment (*SI Appendix*, Fig. S2*C*). These data are consistent with no substantive PSKH1 relocalization in the presence of Ca^2+^, although formal examination of PSKH1 subcellular localization awaits availability of suitable tools for immunofluorescence microscopy.

We next validated these interactions using the orthogonal proteomics methods, Bimolecular Complementation Affinity Purification (BiCAP) (Fig. 2*C*) and immunoprecipitation/mass spectrometry (Fig. 2*D*) in HEK293T cells. BiCAP involves N-terminal fusion of PSKH1 with a split Venus, which when dimerized can be immunoprecipitated by GFP Trap enrichment (Fig. 2*A*). This approach independently validated UNC119B as a PSKH1 interactor (Fig. 2*C*). Immunoprecipitation of C-terminally 3C protease cleavable FLAG-tagged PSKH1 and liberation of binders from M2 resin using 3C protease cleavage identified Ca^2+^ sensor proteins of the CREC family (Fig. 2*D*) to which RCN3 belongs, pointing to a broader role for CREC family proteins in binding to PSKH1. Furthermore, like the pseudokinase paralog, PSKH2 (26), PSKH1 was observed to bind HSP90 and the kinase cochaperone, Cdc37 (Fig. 2*D*), consistent with earlier findings (27), collectively suggesting that a conserved mechanism may underlie PSKH1 and PSKH2 stabilization.

### Calmodulin Allosterically Activates, and CREC Proteins Suppress, PSKH1 Catalytic Output.

**Fig. 3.**
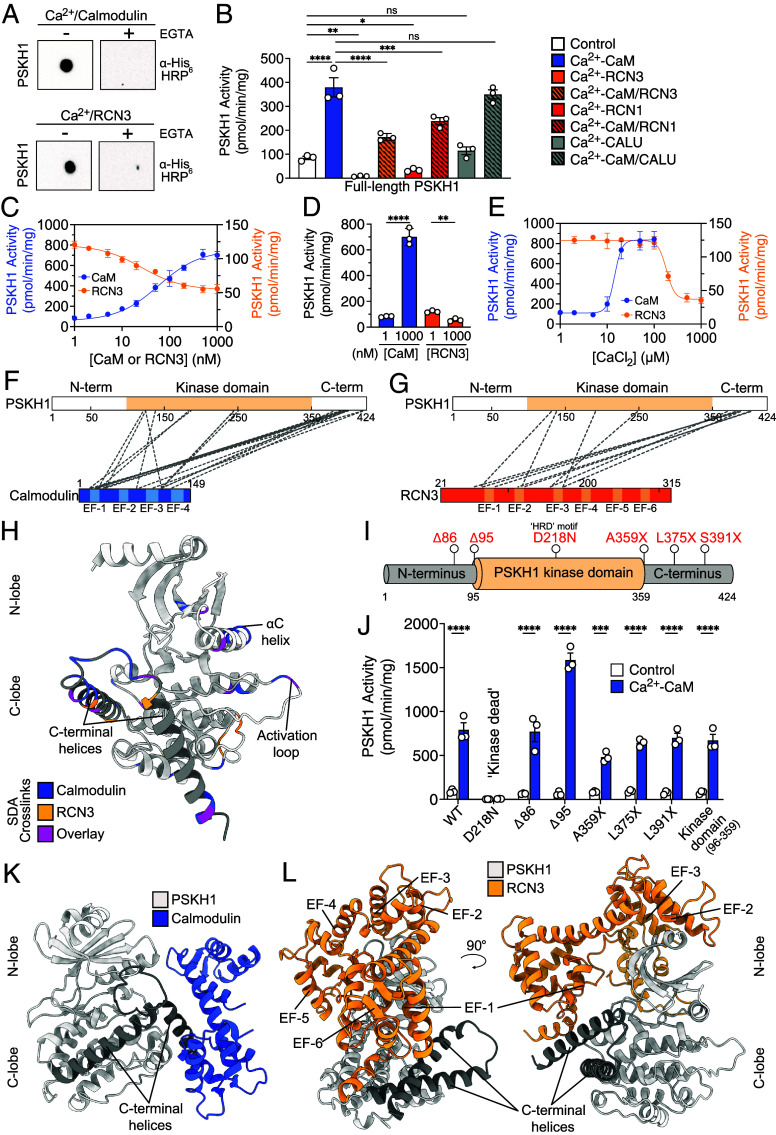
Calcium sensor proteins bind and regulate PSKH1 activity. (*A*) Recombinant PSKH1 interacts with His_6_-Calmodulin (CaM) or His_6_-RCN3 in a Ca^2+^-dependent manner (Ca^2+^: 500 μM) in Far-western blots, with binding abolished in the presence of the Ca^2+^-chelator, EGTA (10 mM). The interaction was probed using anti-His_6_ horseradish peroxidase antibody. Data are representative of two independent replicates. (*B*) Ca^2+^-CaM promotes, and Ca^2+^-Reticulocalbins (RCN1 or 3) antagonize, kinase activity in a radiometric assay of HA-tagged PSKH1 (10 ng). Ca^2+^-Calumenin (CALU) does not modulate PSKH1 kinase activity. CaM, RCN1, RCN3, and CALU were recombinantly expressed and purified (all 1 μM). Individual data points are shown as circles with accompanying mean ± SD; n = 3. Statistical analysis was performed by one-way ANOVA; *, **, ***, and **** signify *P* < 0.1, *P* < 0.01, *P* < 0.001, and *P* < 0.0001, respectively; n.s = nonsignificant. (*C*) Titration of CaM (blue) or RCN3 (orange) (range 0, 1, 2, 5, 10, 20, 50, 100, 200, 500, 1,000 nM) using HA-tagged PSKH1 (10 ng) by radiometric assay. The half-maximal effective concentration (EC_50_) with CaM is 58.7 nM, while the half-maximal inhibitory concentration (IC_50_) with RCN3 is 25.7 nM. Data represent mean ± SD; n = 3. (*D*) Plot of highest and lowest PSKH1 kinase activity from CaM or RCN3 titration. Individual data points are shown as circles with accompanying mean ± SD; n = 3. Statistical analysis was performed by the *t* test; ** and **** signify *P* < 0.01 and *P* < 0.0001, respectively. (*E*) Titration of CaCl_2_ (0, 0.2, 0.5, 1, 2, 5, 10, 20, 50, 100, and 1,000 μM) with CaM (1 μM; blue) or RCN3 (1 μM; orange) using HA-tagged PSKH1 (10 ng) by radiometric assay. The EC_50_ of PSKH1 and CaM by Ca^2+^ is 14.3 μM, while the IC_50_ of PSKH1 and RCN3 by Ca^2+^ is 183 μM. Data represent mean ± SD; n = 3. (*F* and *G*) NHS-Diazirine (SDA; 3.9 Å spacer) chemical crosslinking mass spectrometry of recombinant PSKH1 with Calmodulin (*F*) and RCN3 (*G*). PSKH1 kinase domain is highlighted in beige, Calmodulin in Blue, RCN3 in orange, and crosslinks as gray dashes. The position of each EF-hand motif is annotated on Calmodulin and RCN3. (*H*) AlphaFold model of PSKH1. PSKH1 kinase domain is colored gray and the C-terminal flanking helices in dark gray. Due to very low confidence, the region N-terminal of the kinase domain has been omitted. SDA crosslinks for CaM are colored blue, RCN3 are colored orange and those that overlap are in purple. (*I*) Domain architecture of PSKH1, where individual mutations are annotated in red. N-terminal truncations are denoted by the number of residues deleted (Δ) and C-terminal truncations by the position of the introduced stop (X). (*J*) Radiometric assay of HA-tagged PSKH1 WT and various truncation mutants (10 ng) in the absence (white) and presence of 100 μM CaCl_2_ and 1 μM CaM (blue). Data represent mean ± SD; n = 3. Statistical analysis was performed by two-way ANOVA; **8 and **** signify *P* < 0.001 and *P* < 0.0001, respectively. (*K* and *L*) AlphaFold3 models of PSKH1 and CaM (*K*; colored blue) or RCN3 (*L*: colored orange). PSKH1 kinase domain is colored gray and the C-terminal flanking helices in dark gray. Due to very low confidence, the region N-terminal of the PSKH1 kinase domain has been omitted. EF-hands of RCN3 are annotated. Predicted aligned error (PAE) plots are shown in *SI Appendix*, Fig. S4 *A* and *B*.

Recently, we established that Calmodulin directly bound CHK2—another CAMK family kinase that, like PSKH1, lacks a canonical Calmodulin binding motif—via its kinase domain to suppress CHK2 catalytic activity (5). While Calmodulin was not observed in our PSKH1 interactome studies, secretory pathway Ca^2+^ sensor proteins of the CREC family were proximal to and bound PSKH1 in HEL and HEK293T cells, respectively, leading us to further investigate the interaction of Ca^2+^ sensor proteins with PSKH1. First, we examined the direct binding of Calmodulin and RCN3 to PSKH1, as observed in our PSKH1 proximitome (Fig. 2*B*), using recombinant proteins in a Far Western assay (Fig. 3*A*). These studies verified Calmodulin and RCN3 binding to PSKH1 in a Ca^2+^-dependent manner that was negated by the presence of the Ca^2+^ chelator, ethylene glycol-bis (β-aminoethyl ether)-N,N,N′,N′-tetraacetic acid (EGTA), indicating that a Ca^2+^-mediated conformation change in Calmodulin and a transition to a folded RCN3 structure were crucial to PSKH1 interaction. We next sought to determine the impact of Ca^2+^ sensor protein binding on PSKH1’s catalytic activity in our radiometric assay. Wild-type PSKH1 exhibited robust catalytic activity as measured by ADR1 peptide phosphorylation, and this activity was promoted by Calmodulin ~10-fold and suppressed by RCN3 ~twofold. Calmodulin activation of PSKH1 activity was reduced in the presence of RCN3, indicating that the two Ca^2+^ sensors compete as regulators of PSKH1 kinase activity (Fig. 3*B*). We then examined whether two additional CREC family members identified as PSKH1 binders in HEK293T PSKH1 immunoprecipitates, Reticulocalbin-1 (RCN1) and Calumenin, impacted PSKH1 kinase activity. Using our radiometric assay, RCN1 suppressed PSKH1 catalytic activity to a similar extent as RCN3 and also exhibited a reduction in Calmodulin activation when combined, while Calumenin showed no such effect (Fig. 3*B*). This suggests that RCN1 and RCN3 bind and inhibit PSKH1 via common regulatory mechanisms. We next determined the binding affinity of Calmodulin and RCN3 to PSKH1 by titrating each Ca^2+^ sensor (0, 1, 2, 5, 10, 20, 50, 100, 200, 500, 1,000 nM) in our radiometric assay. These titrations show a half-maximal activation (EC_50_) of PSKH1 by Calmodulin at 58.7 nM and a half-maximal inhibition (IC_50_) of PSKH1 by RCN3 at 25.7 nM (Fig. 3 *C* and *D*). Despite the absence of a canonical Calmodulin binding sequence in PSKH1, the affinity for Calmodulin is in the nanomolar range, like for other CAMK family members (28), with a twofold higher affinity for RCN3 rationalizing why RCN3 can compete with Calmodulin to suppress PSKH1 activation in vitro (Fig. 3*B*). Further, we sought to determine the Ca^2+^ dependence of Calmodulin and RCN3 with PSKH1 by titrating Ca^2+^ up to 1 mM in our radiometric assay. These titrations show an EC_50_ of PSKH1 by Calmodulin at 14.3 μM Ca^2+^ and an IC_50_ of PSKH1 by RCN3 at 183 μM Ca^2+^ (Fig. 3*E*), which are values typical for Calmodulin (7), yet lower than the previously reported millimolar values for RCN3 (29, 30). Importantly, the 10-fold difference in Ca^2+^ dependence supports the notion that these Ca^2+^ sensors operate mutually exclusively as sensors of extremes of Ca^2+^ flux.

Because PSKH1 lacks a conventional Calmodulin binding motif typified by a hydrophobic anchor residue and 2 to 3 basic residues, and CREC sensors are yet to be ascribed roles in kinase regulation, we next sought to define the specific binding interfaces with PSKH1 using crosslinking mass spectrometry and the photoactivatable crosslinker, succinimidyl 4,4′-azipentanoate (SDA; also known as N-Hydroxysuccinimide (NHS)-Diazirine) (Fig. 3 *F* and *G*). SDA is a bifunctional reagent that crosslinks two protein sites via an NHS ester that reacts with primary amines and a diazirine moiety that is coupled to proximal protein sites upon UV light exposure (31). Calmodulin and RCN3 exhibited similar patterns of interaction, with crosslinks to the central kinase domain and the C-terminal flanking region of PSKH1. Unlike the four EF-hand motif protein, Calmodulin, RCN3 is predicted to harbor six EF-hand motifs. Of these, the N-terminal three EF-hand motifs interacted with PSKH1, even though studies of other Reticulocalbins suggest the three C-terminal EF-hand motifs are capable of adopting globular structures in the presence of Ca^2+^ (32). Mapping the crosslinks to an AlphaFold model of PSKH1 revealed a bidentate interaction of Calmodulin and RCN3 with the N- and C-lobes of PSKH1, centered on the regulatory αC helix and activation loop (Fig. 3*H*). Further, the model predicts that the C-terminal flanking region of PSKH1 forms two α-helices that are positioned adjacent to the activation loop and substrate binding pocket, consistent with the observed crosslinks to this region.

To further investigate whether the regions flanking the core kinase domain contribute to Calmodulin-mediated activation of PSKH1 activity, we prepared a series of truncated HA-tagged PSKH1 proteins and examined their amenability to activation by Calmodulin (Fig. 3*I*). As expected, the kinase-dead PSKH1 mutant, D218N, did not exhibit activity in the presence or absence of Calmodulin, while all truncation mutants—including deletion of both N- and C-terminal regions flanking the core kinase domain—were activated to a similar extent upon addition of Calmodulin, except for an even greater activation of the mutant lacking the N-terminal 95 amino acids (Fig. 3*J*). However, this apparent increase in activation is likely attributable to the higher level of expression observed for the PSKH1 Δ95 truncation relative to wild-type (*SI Appendix*, Fig. S3). Conversely, the apparent reduced activation of the C-terminal truncations is likely attributable to a lower level of expression relative to wild-type (*SI Appendix*, Fig. S3). Despite expression differences, these data demonstrate that Calmodulin binding to the core PSKH1 kinase domain is sufficient for activation, which is supported by the spatial arrangement of the Calmodulin crosslinks to the PSKH1 kinase domain within our AlphaFold model (Fig. 3*H*). Activation through direct engagement of the PSKH1 kinase domain is distinct from the conventional mechanism by which Calmodulin activates kinases, which relies on Calmodulin sequestration of a sequence C-terminal to the kinase domain to relieve autoinhibition (5, 33–35). To visualize these interactions, we generated AlphaFold models of PSKH1 with Calmodulin (Fig. 3*K* and *SI Appendix*, Fig. S4*A*) or RCN3 (Fig. 3*L* and *SI Appendix*, Fig. S4*B*). Both complex models showed each Ca^2+^ sensor interacting with the core PSKH1 kinase domain and the flanking C-terminal region, which albeit modelled with lower confidence, corresponds with crosslinks identified between PSKH1 and Calmodulin (Fig. 3*F*) and the crosslinking for RCN3 (Fig. 3*G*). Notably, in the AlphaFold model with RCN3 only EF-hands 1 to 3 engage PSKH1, while EF-hands 4 to 6 face away from the kinase domain, suggesting a structural, rather than protein interaction, function for the C-terminal EF-hands. Greater insight into the binding epitope of each Ca^2+^ sensor and possible regulatory roles of the C-terminal PSKH1 helices awaits experimental structure determination.

### PSKH1 Is Allosterically Activated by UNC119B.

**Fig. 4.**
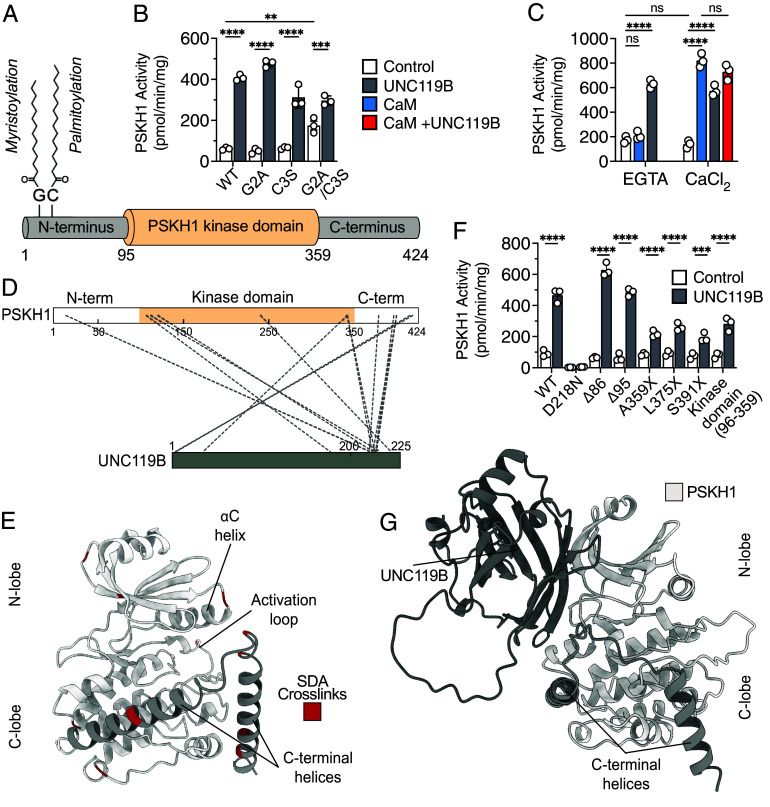
UNC119B binds and activates the PSKH1 kinase domain independent of PSKH1 lipidation. (*A*) Domain architecture of PSKH1, where the myristoyl and palmitoyl groups at positions 2 and 3 are annotated. (*B*) Radiometric assay of HA-tagged PSKH1 WT, G2A, C3S, and a double G2A/C3S mutant (10 ng) in the absence (white) and presence of UNC119B (1 μM; gray). Individual data points are plotted as circles alongside mean ± SD; n = 3. Statistical analysis was performed by two-way ANOVA; **, ***, and **** signify *P* < 0.01, *P* < 0.001, and *P* < 0.0001, respectively. (*C*) Radiometric assay of HA-tagged PSKH1 WT with CaM (1 μM; blue) or UNC119B (1 μM; gray) individually and CaM-UNC119B combined (1 μM each; red), in the presence of EGTA (1 mM) or CaCl_2_ (100 μM). Data represent mean ± SD; n = 3. Statistical analysis was performed by two-way ANOVA; **** signifies *P* < 0.0001; n.s = nonsignificant. (*D*) SDA chemical crosslinking mass spectrometry of PSKH1 and UNC119B. PSKH1 kinase domain is highlighted in beige, UNC119B in dark gray, and crosslinks as gray dashes. (*E*) AlphaFold model of PSKH1. SDA crosslinks from *D* are mapped onto the cartoon structure in maroon. The PSKH1 kinase domain is colored gray and the C-terminal flanking helices in dark gray. (*F*) Radiometric assay of HA-tagged PSKH1 WT and various truncation mutants (10 ng) in the absence (white) and presence of UNC119B (1 μM; gray). N-terminal truncations are denoted by the number of residues deleted (Δ) and C-terminal truncations by the position of the introduced stop (X), as per Fig. 3*J*. Data represent mean ± SD; n = 3. Statistical analysis was performed by two-way ANOVA; *** and **** signify *P* < 0.001 and *P* < 0.0001, respectively. (*G*) AlphaFold3 model of the PSKH1 complex with UNC119B (charcoal). PSKH1 kinase domain is colored gray and the C-terminal flanking helices in dark gray. PAE plot is shown in *SI Appendix*, Fig. S4*C*.

We observed PSKH1 proximity to and interaction with the adaptor protein, UNC119B (Fig. 2 *B* and *C*). This interaction drew our attention for several reasons: PSKH1 is myristoylated and palmitoylated at its N terminus (4) (Fig. 4*A*), and UNC119B is known to function as an adaptor for myristoylated cargo proteins (17, 36–38); UNC119B was previously reported to promote activation of other kinases (18); like PSKH1 (5), UNC119B has been implicated in ciliary organization (15, 16); and, last, UNC119B interacts with PSKH1’s pseudokinase paralog, PSKH2, which exhibits high amino acid identity to PSKH1 within the kinase domain, despite its catalytic inactivity (26, 39). We first examined whether UNC119B influenced PSKH1 kinase activity in our radiometric assay (Fig. 4*B*) revealing, unexpectedly, that UNC119B promoted PSKH1 catalytic activity. Mutation of the predicted binding site of UNC119B on PSKH1, the myristoylated Gly2, the adjacent palmitoylation site, Cys3, or both together, did not block UNC119B activation of PSKH1 and instead led to elevated basal catalytic activity (Fig. 4*B*). These assays revealed that UNC119B binds independently of PSKH1 N-terminal lipidation, suggesting an unconventional mode of allosteric kinase interaction. Additionally, using our radiometric assay, we found that UNC119B activation of PSKH1 is Ca^2+^ independent, which contrasts the requirement of Ca^2+^ for Calmodulin activation of PSKH1 (Fig. 4*C*). Notably, the allosteric activation of PSKH1 by Calmodulin and UNC119B is not additive when both activating proteins are combined (Fig. 4*C*), suggesting that UNC119B and Calmodulin may act on PSKH1 via overlapping binding sites.

To further investigate, we mapped where UNC119B binds PSKH1 using crosslinking mass spectrometry (Fig. 4 *D* and *E*), identifying the UNC119B C-terminal region and the N-lobe of the kinase domain and flanking region C-terminal to the kinase domain of PSKH1 as the principal interaction sites. Truncation analysis supported our crosslinking findings (Fig. 4*F*): The region N-terminal to the kinase domain could be deleted without compromising PSKH1 activity, whereas deletion of the flanking region C-terminal to the kinase domain suppressed UNC119B activation of PSKH1. Moreover, deletion of the N- and C-terminal flanks of the kinase domain together did not further compromise UNC119B activation of PSKH1. These data indicate that UNC119B binds and activates the kinase domain of PSKH1, with this interaction augmented by the C-terminal flank of the kinase domain. Interestingly, AlphaFold models of PSKH1 in complex with Calmodulin (Fig. 3*K* and *SI Appendix*, Fig. S4*A*) and UNC119B (Fig. 4*G* and *SI Appendix*, Fig. S4*C*) suggest very different modes of interaction with PSKH1, albeit with each involving kinase N-lobe and C-terminal flanking helices engagement by each interactor. A more precise understanding of how each Ca^2+^ sensor and UNC119B engage PSKH1 awaits experimental structure determination.

## Discussion

The biological function of the PSKH1 kinase and the mechanisms underlying its regulation have remained underexplored since initial biochemical studies in the early 2000s (4, 6, 40). Only recently was the loss of kinase activity causatively linked to kidney ciliopathies in human patients (3), supporting an earlier attribution of PSKH1’s involvement in heart cilia organization in mice from a mutagenesis screen for coronary heart disease regulators (41). A critical challenge to advancing knowledge of PSKH1 biochemistry has been a lack of protocols for producing recombinant protein and the absence of knowledge of substrates or interacting proteins. Here, we overcame these limitations by establishing PSKH1 expression and purification protocols, a robust radiometric assay to measure catalytic activity, defining the PSKH1 interactome using three complementary methods, and detailing PSKH1’s allosteric regulation by Ca^2+^ sensor proteins and the UNC119B adaptor.

An unexpected finding from our study is the prevalence of Ca^2+^ sensor proteins as interactors and regulators of PSKH1 activity. PSKH1 is an ER-resident protein kinase by virtue of N-terminal lipidation, which would localize PSKH1 to secretory organelles proximal to the CREC family of Ca^2+^ sensors—Cab45 (SDF4), Reticulocalbin (RCN)-1 and -3, Erc55 (RCN2), Calumenin. The CREC proteins are predicted to be localized to the ER lumen owing to an N-terminal targeting sequence and a C-terminal HDEL motif that is thought to maintain CREC proteins in the ER (11). However, the precise subcellular locations of PSKH1 and CREC proteins have not been robustly established experimentally, and detailed exploration awaits the development of suitable, specific reagents. Nonetheless, the observation of CREC proteins in different cell lines in distinct PSKH1 proximitome and interactome experiments indicates their proximity in cells, and the prospect of CREC proteins physiologically modulating PSKH1 activity. Whether CREC proteins have functions in kinase regulation had been an open question, which makes our finding that RCN1 and RCN3 could suppress in vitro PSKH1 kinase activity of great interest. Moreover, RCN3 binding to PSKH1 occurred via an interface akin to that of Calmodulin, despite the latter enhancing activity, whereas RCN3 was inhibitory. PSKH1 regulation through binding of Calmodulin and CREC proteins directly to the kinase domain is reminiscent of our recent finding that Calmodulin can suppress CHK2 kinase activity by binding the kinase domain surface via an interface centered in the activation loop (5).

**Fig. 5.**
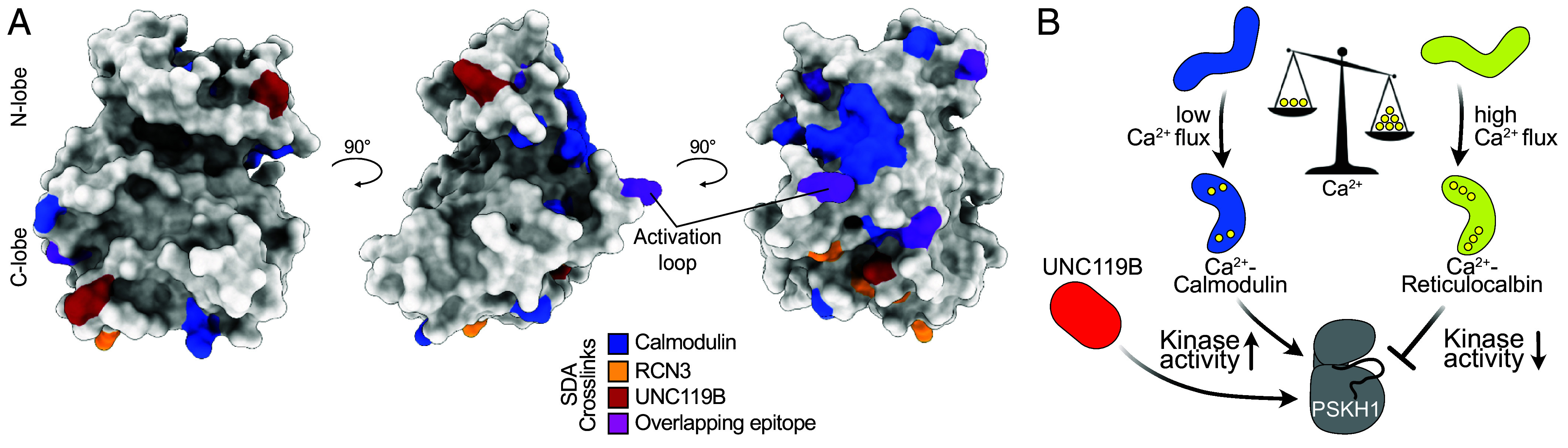
Allosteric binders converge on common sites on PSKH1. (*A*) Orthogonal views of PSKH1 with sites bound by Calmodulin (blue), RCN3 (yellow), UNC119B (red), and all interactors (purple) highlighted. (*B*) Schematic overview of positive and negative allosteric binding interactions identified in this work. Extremes in Ca^2+^ flux are predicted to dictate whether PSKH1 is activated (Calmodulin; low Ca^2+^) or inhibited (RCN1 or RCN3; high Ca^2+^) by different Ca^2+^ sensor proteins. Like Ca^2+^ sensor proteins, UNC119B binds directly to the PSKH1 kinase domain, but allosterically activates kinase activity in a Ca^2+^ and lipidation-independent manner.

Our observations expand the repertoire of kinase regulation modes conferred by Calmodulin and other Ca^2+^ sensor proteins (Fig. 5*A*). Perhaps accounting for why there is such diversity in the cellular complement of Ca^2+^ sensing proteins, not only are CREC proteins localized to the secretory machinery like PSKH1 but also regulated distinctly to the archetypal Ca^2+^ sensor, Calmodulin. Calmodulin undergoes a structural transition on Ca^2+^ binding (10) with typical K_d_ values reported in the 0.5 to 5 μM range (7), comparable to the half-maximal effective concentration (EC_50_) of 14 μM for Ca^2+^ that we observed herein in Calmodulin activation of PSKH1. CREC proteins undergo an unfolded-to-folded transition on Ca^2+^ binding (13, 14), which has been reported to occur at vastly higher Ca^2+^ concentrations than for Calmodulin, with K_d_ values in the millimolar range (29, 30). Like for Calmodulin, our data suggest a broader range of CREC protein affinities for Ca^2+^ than has been appreciated to date, with a half-maximal inhibitory concentration (IC_50_) of 183 μM for Ca^2+^ binding in RCN3 inhibition of PSKH1 activity. Intracellular Ca^2+^ flux is known to range from 100 nM under basal conditions to 1 mM in different physiological contexts and cell types (42). As a result, extremes in [Ca^2+^] could govern whether Calmodulin or CREC family proteins predominate as PSKH1 regulators. While Calmodulin’s transition to a PSKH1-binding competent form requires a modest elevation in Ca^2+^ concentration from the basal intracellular concentration of 0.1 μM, substantively higher and ER-localized flux would be required to license CREC proteins to bind and suppress PSKH1 activity. By extension, we propose a rheostatic model in which the activity of PSKH1 is dictated by proximal Ca^2+^ levels (Fig. 5*B*). In this model, Calmodulin activates PSKH1 at low Ca^2+^, and CREC sensors suppress PSKH1 activity at high Ca^2+^ thus allowing dynamic modulation of PSKH1 catalytic activity depending on the Ca^2+^ flux into cells or release from intracellular stores.

In addition to Ca^2+^ sensing proteins, our interactomics identified the chaperone protein, UNC119B, which is best described in facilitating protein trafficking between intracellular membranes via its binding to N-myristoylated peptides in its cargoes (36, 38, 43, 44). An analogous mode of binding to the myristoylated N terminus of Src family kinases was previously reported to promote kinase activity by relieving lipid occlusion of the kinase active site. Here, we have identified a distinct mode of UNC119B activation of a kinase—where direct binding to the kinase domain, independent of lipidation, promoted kinase activity. Interestingly, UNC119B was identified in our BiCAP mass spectrometry experiments, as an interactor of dimerized PSKH1, raising the possibility that UNC119B binds higher-order assemblies of PSKH1 to promote their kinase activity. Interaction of UNC119 with PSKH1, as well as the pseudokinase paralog, PSKH2, has been previously observed in interactome screens (45–47), although whether these interactions could serve regulatory functions was an open question. Understanding the precise mode of interaction of UNC119B with PSKH1, and whether this differs from the activating interaction with Calmodulin, awaits high-resolution structure determination. However, it is worth noting that crosslinks for each of Calmodulin and UNC119B in our structural mass spectrometry studies map to proximal sites in an AlphaFold model of PSKH1 (Fig. 5). Such an overlap raises the possibility that a regulatory hotspot may exist on the PSKH1 surface that can be allosterically activated, despite the vastly different folds and predicted binding modes of each activator protein. Based on crosslinking mass spectrometry, the C-terminal helices flanking the PSKH1 kinase domain are a common denominator in binding to each interactor. While the disposition of these PSKH1 helices is comparable between Calmodulin and RCN3 in their complex AlphaFold models, and differs in the UNC119B complex model, the C-terminal helices are modeled with low confidence. As a result, further studies are required to establish whether these helices serve a regulatory function in modulating PSKH1 activity and how this relates to their disposition.

Collectively, our study has advanced knowledge of the understudied protein kinase, PSKH1, and identified mechanisms by which kinases can be allosterically regulated by protein interactors, including by the understudied CREC Ca^2+^ sensor family. Our study highlights the potential of CREC Ca^2+^ sensors to complement Calmodulin in regulating kinase activity depending on extremes of Ca^2+^ flux. Furthermore, our findings highlight the complexity of mechanisms that can be integrated by kinases to regulate their activities. The applicability of the modes of Ca^2+^ sensor and UNC119B regulation of PSKH1 identified herein to other kinases, including those that remain understudied, awaits careful biochemical examination.

## Materials and Methods

Complete descriptions of experimental procedures are included in *SI Appendix*. Brief summaries of procedures are included as follows.

### Recombinant Protein Expression and Purification.

Full-length human PSKH1 (Uniprot P11801) and full-length human UNC119B (Uniprot A6NIH7) were expressed and purified from Expi*Sf*9 insect cells via the Bac-to-Bac system (Invitrogen) using established procedures (3) and GST affinity chromatography, respectively. Human Calmodulin (Uniprot P0DP23), RCN1 (Uniprot O15293; gene kindly gifted by Prof. Naoto Yonezawa, Chiba University), RCN3 (Uniprot Q96D15), and Calumenin (CALU; Uniprot O43852) were expressed and purified from *Escherichia coli* BL21-Codon Plus (DE3)-RIL using NiNTA or GST affinity chromatography and gel filtration.

### Peptide Array.

Positional scanning peptide array experiments and analyses were performed as reported previously (21) using purified recombinant full-length PSKH1 from Expi*Sf*9 insect cells.

### Mass Spectrometry.

For TurboID proximity labeling, PSKH1 was subcloned into the mammalian expression vector, pFTRE3G PGK puro (48), as an in-frame fusion with a C-terminal FLAG tag and TurboID fusion, and stably transduced into HEL cells (HEL 92.1.7; sourced from American Type Culture Collection). Briefly, expression was induced overnight with doxycycline (100 ng/mL) and then treated with biotin (500 µM) for 10 min prior to cell harvest. In parallel TurboID experiments, during biotin treatment, ionomycin was included to induce Ca^2+^ influx. Biotinylated proteins were captured from lysates using high-capacity Streptavidin (ThermoFisher Scientific), digested with trypsin (Sigma), and then analyzed on a timsTOF Pro (Bruker, Billerica, MA). For BiCAP experiments, PSKH1 was subcloned into the mammalian expression vector, pDEST, as fusions to two half venus protein fragments (V1 and V2), and transient transfected into HEK293T cells. BiCAP analysis of the PSKH1 dimer was performed as previously described (49, 50). For FLAG-IP experiments, PSKH1 was subcloned into the mammalian expression vector, pcDNA3, as an in-frame fusion with a C-terminal, 3C protease-cleavable FLAG epitope tag, and transient transfected into HEK293T cells. Briefly, lysates were incubated with anti-FLAG G1 Affinity Resin (GenScript) and proteins were eluted using 3C protease. Following tryptic digest, proteins were analyzed using a Thermo QExactive mass spectrometer (ThermoFisher Scientific).

### In Vitro Kinase Assays.

Full-length PSKH1 with a C-terminal HA-tag (subcloned into pcDNA3.1) were transiently expressed in HEK293T cells grown in Dulbecco's Modified Eagle Medium (ThermoFisher Scientific) media, supplemented with 8% (v/v) Fetal Calf Serum (ThermoFisher Scientific) at 37 °C with 5% CO_2_. Following pulldown with anti-HA agarose (Sigma), PSKH1 activity was determined by measuring the transfer of radiolabeled phosphate from [γ-^32^P]-ATP to a synthetic peptide substrate (ADR1; LKKLTRRASFSGQ; Genscript), as described before (3).

### Chemical Crosslinking Mass Spectrometry.

Recombinant full-length PSKH1 and either recombinant Calmodulin, RCN3, or UNC119B (1:2 molar ratio) was mixed with SDA (NHS-Diazirine; 1 mg/mL; ThermoFisher Scientific), incubated in the dark for 30 min at room temperature to react the NHS-ester group, and then the diazirine moiety was photoactivated using ultraviolet light irradiation (UVP CL-1000L UV cross-linker) at 365 nm. Crosslinked species were resolved by reducing sodium dodecyl sulfate–polyacrylamide gel electrophoresis gel electrophoresis (Bio-Rad), excised by scalpel, digested with trypsin (ThermoFisher Scientific), and then analyzed on Orbitrap Eclipse Tribrid mass spectrometer.

## Supplementary Material

Appendix 01 (PDF)

## Data Availability

All reagents are available under material transfer agreement. TurboID, BiCAP, and FLAG-IP interactome mass spectrometry proteomics data have been deposited to the ProteomeXchange Consortium via the PRIDE (51) partner repository with the dataset identifiers PXD056543, PXD058954, and PXD055768. SDA crosslinking mass spectrometry data have been deposited to the ProteomeXchange Consortium via jPOST (52) with accession numbers: JPST003399 and PXD056468 for PSKH1:Calmodulin; JPST003400 and PXD056476 for PSKH1:UNC119B; and JPST003401 and PXD056478 for PSKH1:RCN3.
