# Supplementary material for: PSKH1 kinase activity is differentially modulated via allosteric binding of Ca2+ sensor proteins
Source: Proc Natl Acad Sci U S A. 2025 Feb 18;122(8):e2420961122. doi: 10.1073/pnas.2420961122 (PMC11873932; doi:10.1073/pnas.2420961122)
Supplement: Supplementary file 1 — Appendix 01 (PDF) [file pnas.2420961122.sapp.pdf]

PNAS

Supporting Information for
PSKH1 kinase activity is differentially modulated via allosteric binding of Ca^{2+} sensor proteins

Christopher R. Horne[1,2,3*], Toby A. Dite[1,2], Samuel N. Young[1], Lucy J. Mather[1], Laura F. Dagley[1,2], Jared L. Johnson[4,5,6], Tomer M. Yaron-Barir[4,7,8], Emily M. Huntsman[4,7], Leonard A. Daly[9,10], Dominic P. Byrne[9], Antonia L. Cadell[11], Boaz H. Ng[11], Jumana Yousef[1,2], Dylan H. Multari[1,2], Lianju Shen[1], Luke M. McAloon[3,12,13], Gerard Manning[14], Mark A. Febbraio[3], Anthony R. Means[15], Lewis C. Cantley[4,5,6], Maria C. Tanzer[1,2], David R. Croucher[11,16], Claire E. Eyers[9,10], Patrick A. Eyers[9], John W. Scott[3,13,17,*†], James M. Murphy[1,2,3,*†]

Christopher Horne, John Scott, James Murphy
Email: horne.c@wehi.edu.au (CRH), john.scott@monash.edu (JWS) or jamesm@wehi.edu.au (JMM)

This PDF file includes:

Supplementary Information Text
Figures S1 to S4
Supplementary Information References

Supplementary Information Text

Materials and Methods

Expression constructs

The gene coding for full-length human PSKH1 (Uniprot P11801) was synthesised by Gene Universal (Delaware, USA) and subcloned into a mammalian expression vector, pcDNA3.1(-), using the restriction sites XhoI and HindIII with a C-terminal HA tag. PSKH1 variants were introduced into the wild-type template using NEBuilder HiFi DNA Assembly (New England Biolabs). PSKH1 was also subcloned into the insect expression vector, pFastBac GST-GFP, as an in-frame fusion with a TEV protease-cleavable N-terminal GST tag and 3C- protease-cleavable N-terminal GFP tag using the restriction sites BamHI and EcoRI, as described previously (1). For TurboID experiments, PSKH1 was subcloned into the mammalian expression vector, pFTRE3G PGK puro (2), as an in-frame fusion with a C-terminal FLAG tag and TurboID fusion using the restriction sites BamHI and NheI. The vector encoding TurboID was sourced from Addgene (#107171) (3) and kindly provided by Prof. Peter Mace (University of Otago) with the generous permission of Prof. Alice Ting (Stanford University). For BiCAP experiments, PSKH1 was first subcloned into the Gateway Cloning donor vector, pDONR221. The two BiCAP expression vectors were subsequently generated by recombination of the PSKH1 sequence into the pDEST-V1-ORF and pDEST-V2-ORF destination vectors using Gateway Cloning. For FLAG-IP experiments, PSKH1 was subcloned into a pcDNA3 backbone vector as an in-frame fusion with a C-terminal, 3C protease-cleavable FLAG epitope tag using restriction cloning. The gene coding for full-length Calmodulin (CaM; Uniprot P0DP23) was synthesised by IDT (Iowa, USA) as a gBlock and subcloned into the bacterial expression vector, pPROEX Htb (Life Technologies), as an in-frame fusion with a TEV protease-cleavable N-terminal hexahistidine tag using the restriction sites BamHI and NotI. The gene coding for full-length Reticulocalbin-3 (RCN3; Uniprot Q96D15) was synthesised by IDT (Iowa, USA) as a gBlock and subcloned into the bacterial expression vector, pPROEX Htb (Life Technologies), as an in-frame fusion with a TEV protease-cleavable N-terminal hexahistidine tag using the restriction sites BamHI and EcoRI. The gene coding for full-length UNC119B (Uniprot A6NIH7) was synthesised by GenScript (New Jersey, USA) and subcloned into the insect expression vector, pFastBac GST, as an in-frame fusion with a TEV protease-cleavable N-terminal GST tag using the restriction sites BamHI and EcoRI. The gene coding for full-length reticulocalbin-1 (RCN1; Uniprot O15293) was a kind gift from Prof. Naoto Yonezawa (Chiba University) (4). The gene encoding full-length Calumenin (CALU; Uniprot O43852) was synthesised by GenScript (New Jersey, USA) and subcloned into the bacterial expression vector, pGEX-2T-TEV (5), as an in-frame fusion with a TEV protease-cleavable N-terminal GST tag using the restriction sites BamHI and EcoRI. Insert sequences were verified by Sanger sequencing (AGRF, VIC, Australia). The gene encoding full-length PSKH2 (Uniprot Q96QS6; IMAGE clone BC126180) (6), was subcloned into the insect expression vector, pFastBac GST-GFP (1), as an in-frame fusion with a TEV protease-cleavable N-terminal GST tag and 3C- protease-cleavable N-terminal GFP tag using the restriction sites BamHI and EcoRI.

Recombinant Expression and Purification

PSKH1, PSKH2 and UNC119B, each harboring a N-terminal GST tag, were expressed and purified from Expi*Sf*9 insect cells. Briefly, the bacmid was prepared in DH10MultiBac *Escherichia coli* (ATG Biosynthetics) from a pFastBac GST-GFP vector containing PSKH1 or PSKH2 as a BamHI-EcoRI insert. *Sf*21 insect cells (Merck) were cultured in Insect-XPRESS (Lonza) media. Each bacmid (1μg) was introduced into 0.9 × 10^6 *Sf*21 cells by Cellfectin II (Thermo Fisher Scientific) mediated transfection in six-well plates using the Bac-to-Bac protocol (Thermo Fisher Scientific), as detailed elsewhere (7). After 4 days of static incubation at 27 °C in a humidified incubator, the resulting P1 baculovirus was harvested and added to 50 mL *Sf*21 cells at 0.5 × 10^6 cells/mL density at 4% v/v, which were shaken at 27 °C, 130 rpm. The cell density was monitored daily using a haemocytometer slide and maintained at 0.5–3.0 × 10^6 cells/mL by diluting with fresh Insect-XPRESS medium when necessary, until growth arrest (defined as a cell density less than the twice the cell count 1 day prior). Approximately 24 h after growth arrest was recorded, the P2 baculovirus was harvested by collecting the supernatant after pelleting the cells at 500 × *g* for 5 min. P2

baculovirus (3 mL) was added to 0.5 L Expi*Sf*9 cells, seeded at 6 × 10^6 cells/mL into 2.8 L Fernbach flasks using Expi*Sf* CD Medium (Thermo Fisher Scientific). Expi*Sf*9 cells were harvested 72 h post transduction at 500 ×*g* and pellets snap frozen in liquid N_2 and stored at −80 °C.

CaM, RCN3, RCN1 and CALU were expressed in *E. coli* BL21-CodonPlus-RIL (Agilent) cells cultured in Super Broth supplemented with ampicillin (100 µg/mL) at 37 °C with shaking at 220 rpm to an OD_{600} of ~0.6–0.8. Protein expression was induced by the addition of isopropyl β-D-1-thiogalactopyranoside (IPTG; 250 µM) after the incubator temperature was lowered to 18 °C; shaking incubation was continued overnight. Bacterial cells were harvested by centrifugation at 2000 ×*g* and pellets snap frozen in liquid N_2 and stored at −80 °C.

PSKH1, PSKH2, UNC119B and CALU cell pellets were resuspended in GST buffer (20 mM HEPES pH 7.5, 200 mM NaCl, 10% v/v glycerol), supplemented with cOmplete Protease Inhibitor (Roche) and 0.5 mM Bond-Breaker TCEP [tris-(2-carboxyethyl)phosphine] (Thermo Fisher Scientific) and were lysed by sonication, before the lysate was clarified by centrifugation (40,000 ×*g*, 45 min, 4 °C). The proteins were maintained at 4 °C throughout purification. The clarified lysate was incubated with Glutathione Xpure Agarose resin (UBPBio) pre-equilibrated in GST buffer for >1 h on rollers at 4 °C, before the beads were pelleted at 500 ×*g* and washed extensively with GST buffer. PSKH1 was further purified by cleaving the GST tag (on resin) with recombinant His_6-TEV protease overnight at 4 °C. The supernatant was then syringe filtered (0.2 µm), spin concentrated (30 kDa MWCO; Millipore), and then loaded onto a HiLoad 16/160 Superdex 200 pg column (Cytiva) pre-equilibrated with SEC buffer (20 mM HEPES pH 7.5, 200 mM NaCl, 5% v/v glycerol). Purified fractions, as assessed following resolution by reducing StainFree SDS-PAGE gel electrophoresis (Bio-Rad), were pooled, spin concentrated, aliquoted, and snap frozen in liquid N_2 for storage at −80 °C.

CaM, RCN3 and RCN1 cell pellets were resuspended in Ni-NTA buffer (20 mM HEPES pH 7.5, 200 mM NaCl, 10% v/v glycerol), supplemented with 10 mM imidazole (pH 8.0), EDTA-free cOmplete Protease Inhibitor (Roche) and 0.5 mM Bond-Breaker TCEP and were lysed by sonication, before the lysate was clarified by centrifugation (40,000 × *g*, 45 min, 4 °C). The clarified lysate was incubated with Ni-NTA resin (Roche) pre-equilibrated in Ni-NTA buffer with 5 mM imidazole for >1 h on rollers at 4 °C, before the beads were pelleted at 500 × *g* and washed extensively with Ni-NTA buffer containing 35 mM imidazole. The proteins were eluted in Ni-NTA buffer containing 250 mM imidazole. The eluate was further purified by cleaving the His_6 tag using recombinant His_6-TEV protease (for applications where the absence of His_6 tag was desirable), before dialysing in GST buffer and addition of Ni-NTA resin to eliminate any uncleaved material and the TEV protease. TEV protease-cleaved samples were spin concentrated (10 kDa MWCO; Millipore), before being loaded onto a HiLoad 16/160 Superdex 200 pg column (Cytiva) pre-equilibrated with SEC buffer (20 mM HEPES pH 7.5, 200 mM NaCl, 5% v/v glycerol). Purified fractions, as assessed following resolution by reducing StainFree SDS-PAGE gel electrophoresis (Bio-Rad), were pooled and spin concentrated, aliquoted, and snap frozen in liquid N_2 for storage at −80 °C.

Peptide Array

Positional scanning peptide array experiments and analyses were performed as reported previously (8). Briefly, recombinant PSKH1 was added to a 384-well plate containing 50 µM peptide substrate library mixtures (Anaspec, AS-62017-1 and AS-62335). The reaction was initiated with the addition of 50 µM ATP (50 µCi mL^{-1} γ-^{32}P-ATP, Perkin-Elmer) and incubated for 90 min at 30°C in 50 mM HEPES pH 7.4, 10 mM $MgCl_2$, in the presence or absence of 0.2 µM Calmodulin, 400 µM $CaCl_2$ (SignalChem catalog # C02-39B-500 calcium/Calmodulin solution); no differences were evident between conditions. Solutions were spotted onto Streptavidin-conjugated membranes (Promega, V2861) via the C-terminal biotin on the peptides upon completion of the reaction, before membrane rinsing and imaging using a Typhoon FLA 7000 phosphorimager (GE), and raw data quantification using ImageQuant (GE) to generate densitometry matrices. Densitometry matrices were column-normalized at all positions by the sum of the 17 randomized amino acids (excluding serine, threonine and cysteine), to obtain position-specific scoring matrices (PSSMs).

Generation of TurboID cell lines and validation

Midiprep DNA of the pFTRE3G PGK Puro vector encoding a PSKH1-TurboID fusion was co-transfected into HEK293T cells (originally sourced from ATCC) with helper plasmids pVSVg and pCMV δR8.2 to generate lentiviral particles using Effectene (Qiagen). HEL cells (HEL 92.1.7; sourced from ATCC) were then stably transduced with the resulting lentivirus and successful transductants selected using puromycin (1.25 µg/mL; StemCell Technologies) as before (2, 9). Transduced HEL cells were cultured in RPMI + 8% FCS at 37 °C and 10% (v/v) CO_2 and were routinely PCR monitored for mycoplasma contamination. Expression was validated by Western Blot using a rat anti-FLAG monoclonal antibody (as per *Western Blot* and *Antibodies* below).

TurboID proximity labelling and mass spectrometry analysis

HEL cells (30 × 10^6 cells) were seeded into T75 flasks and exogene expression induced overnight with 100 ng/mL doxycycline. The following morning, cells were treated with Biotin (500 µM) for 10 mins and washed 6 times with ice-cold PBS by centrifugation (1500 rpm, 5mins. 4°C) to remove excess biotin. In a parallel experiment to determine the effect of Ca^{2+} flux on PSKH1 interactions, ionomycin (1 µM) was added simultaneously with Biotin for 10 mins before cells were washed with ice-cold PBS. Cells were then lysed in buffer (50 mM Tris-HCl pH 7.4, 1% (v/v) Triton X-100, 1 mM PMSF, 1 mM EDTA, 150 mM NaCl, 2 mM Sodium Vanadate, 10 mM NaF) containing a cOmplete protease inhibitor tablet (Roche) and insoluble debris were removed by centrifugation. Biotinylated proteins were captured from lysates (500 µg per replicate) by incubation with 10 µg high-capacity Streptavidin agarose (ThermoFisher Scientific #20359) for 1 hour, rotating at 4°C. The beads were then washed three times with lysis buffer, and three times with PBS + 0.5% SDS before being incubated with 100 µl PBS + 0.5% SDS + 1 mM DTT for 30 minutes at room temperature. The beads were then washed once in 50 mM ammonium bicarbonate + 6 M urea and transferred to Snap Cap Spin Columns (Pierce #69725). Using centrifugation at 1000 ×*g* for 1 min, the beads were washed 5 times 50 mM ammonium bicarbonate + 6 M urea, 5 times with PBS, and 3 times with H_2O. Proteins were then digested on-bead for 16 hours, at 37°C using 1 µg trypsin (Sigma #EMS0004) in 50 mM ammonium bicarbonate. Peptides were then collected into new tubes by centrifugation. The collected peptides were lyophilized to dryness using a CentriVap (Labconco), before reconstituting in 30 µL 0.1% v/v formic acid/2% v/v acetonitrile ready for mass spectrometry analysis. Peptides were separated by reverse-phase chromatography on a C18 fused silica column (inner diameter 75 µm, OD 360 µm × 15 cm length, 1.6 µm C18 beads) packed into an emitter tip (IonOpticks, Fitzroy, Victoria, Australia), using a nano-flow HPLC (M-class, Waters, Milford, Massachusetts, USA) coupled to a timsTOF Pro (Bruker, Billerica, Massachusetts, USA) equipped with a CaptiveSpray source. Peptides were loaded directly onto the column at a constant flow rate of 400 nL/min with buffer A (99.9% Milli-Q water, 0.1% FA) and eluted with a 30-min linear gradient from 2 to 34% buffer B (99.9% ACN, 0.1% FA). The timsTOF Pro (Bruker) was operated in diaPASEF mode using Compass Hystar 5.1. The settings on the TIMS analyzer were as follows: Lock Duty Cycle to 100% with equal accumulation and ramp times of 100 ms, and 1/K0 Start 0.6 V.·/cm2 End 1.6 V·s/cm2, Capillary Voltage 1400V, Dry Gas 3 l/min, Dry Temp 180°C. The DIA methods were set up using the instrument firmware (timsTOF control 2.0.18.0) for data-independent isolation of multiple precursor windows within a single TIMS scan. The method included two windows in each diaPASEF scan, with window placement overlapping the diagonal scan line for doubly and triply charged peptides in the m/z – ion mobility plane across 16 × 25 m/z precursor isolation windows (resulting in 32 windows) defined from m/z 400 to 1,200, with 1 Da overlap, and CID collision energy ramped stepwise from 20 eV at 0.8 V·s/cm2 to 59eV at 1.3 V·s/cm2.Data files were analysed by DIA-NN v1.8.1 software (10). Data were searched against the human Uniprot Reference Proteome with isoforms (downloaded May 2021), with recombinant protein sequences added, as a FASTA digest for library-free search with a strict trypsin specificity allowing up to 2 missed cleavages. The peptide length range was set to 7-30 amino acids. Precursor charge range was set between 1-4, and m/z range of 300-1800. Carbamidomethylation of Cys was set as a fixed modification. Precursor FDR was set to 1% and match between runs was enabled. Data processing and analysis of the DIA-NN output were performed using R software (v. 4.2.1). Protein groups were filtered based on a precursor-level q-value of <1% and a protein group-level q-value of <1%, ensuring that only high-confidence identifications were retained. Additionally,

only proteins identified with proteotypic peptides were considered for downstream analysis. The PG.MaxLFQ values were used to derive normalised protein group abundances. To ensure data quality, protein groups present in at least 50% of replicates in at least one experimental condition were retained for further analysis. This filtering resulted in a final set of 5,062 proteins. Protein intensity values were subsequently log_2-transformed to meet the assumptions of downstream statistical tests. Normalization was carried out using RUVIII-C method (v1.0.19) to reduce unwanted technical variation across samples. Missing values were imputed by drawing random numbers from a normal distribution (with a width parameter of 0.3 and a downshift of 1.8. Principal Component Analysis (PCA) was performed to reduce the dimensionality of the data and identify potential outliers. Differential expression analysis was conducted using the limma R package (v.3.50.0), applying empirical Bayes moderation to improve statistical power. Proteins were deemed significantly differentially expressed if they passed a false discovery rate (FDR) threshold of ≤ 5% after Benjamini–Hochberg (BH) correction. Data visualization was conducted using the ggplot2 R package. Gene Ontology analysis was performed using Metascape (11) with *Homo sapiens* set as the background. Proteins from the TurboID experiment displaying >1.5 fold enrichment with a statistical significance of P<0.05 were used for the analysis.

Bimolecular complementation affinity purification (BiCAP) analysis

BiCAP analysis of the PSKH1 dimer was performed using established protocols (12, 13) . Briefly, $1x10^6$ HEK293T cells were seeded into 10 cm dishes in 10 mL of growth media 24 h prior to transfection. Samples were prepared in quadruplicate. The two BiCAP expression constructs, pDEST-V1-PSKH1 and pDEST-V2-PSKH2, or a control plasmid expressing full length Venus, were transfected with Jetprime Transfection Agent (Polyplus). Lysates were harvested ~20 hours after transfection following visual observation of fluorescence. Affinity purification was performed using GFP-Trap® Magnetic Agarose (ChromoTek) beads and captured proteins were prepared for mass spectrometry analysis using the FASP (filter-aided sample preparation) method (14), with the following modifications. Proteins were eluted from GFP-Trap beads using 100 µL 0.5% w/v SDS in PBS at 60°C for 3 minutes. Proteins were reduced with 10 mM Tris-(2-carboxyethyl) phosphine (TCEP), alkylated with 50 mM iodoacetamide, then digested with 1 µg sequence-grade modified trypsin gold (Promega) in 50 mM ammonium bicarbonate and incubated overnight at 37°C. Peptides were eluted with 50 mM ammonium bicarbonate in two 40 µL sequential washes and acidified in 1% formic acid (FA, final concentration). The collected peptides were lyophilized to dryness using a CentriVap (Labconco), before reconstituting in 10 µL 0.1% v/v formic acid/2% v/v acetonitrile ready for mass spectrometry analysis. Peptides (3 µL) were separated by reverse-phase chromatography on a C18 fused silica column (inner diameter 75 µm, OD 360 µm × 15 cm length, 1.6 µm C18 beads) packed into an emitter tip (IonOpticks, Austalia) using a custom nano-flow HPLC system (Thermo Ultimate 3000 RSLC Nano-LC, PAL systems CTC autosampler). The HPLC was coupled to a timsTOF Pro (Bruker) equipped with a CaptiveSpray source. Peptides were loaded directly onto the column at a constant flow rate of 400 nL/min in diaPASEF mode as described above. Data files were analysed by DIA-NN v1.8.1 software. Data was searched against the human Uniprot Reference Proteome with isoforms (downloaded August 2022), with recombinant protein sequences added, as a FASTA digest for library-free search with a strict trypsin specificity allowing up to 2 missed cleavages. The peptide length range was set to 7-30 amino acids. Precursor charge range was set between 1-4, and m/z range of 300-1800. Carbamidomethylation of Cys was set as a fixed modification. Precursor FDR was set to 1% and match between runs was on. Data processing and analysis of the DIA-NN output were performed using R software (v. 4.2.1). Protein groups were filtered based on a precursor-level q-value of <1% and a protein group-level q-value of <1%, ensuring that only high-confidence identifications were retained. Additionally, only proteins identified with proteotypic peptides were considered for downstream analysis. The PG.MaxLFQ values were used to derive normalised protein group abundances. To ensure data quality, protein groups present in at least 50% of replicates in at least one experimental condition were retained for further analysis. This filtering resulted in a final set of 4,092 proteins. Protein intensity values were subsequently log_2-transformed to meet the assumptions of downstream statistical tests. Normalisation was carried out using the cyclic loess method implemented in the limma R package (v. 3.52.2) to reduce unwanted technical variation across samples. Principal Component Analysis (PCA) was performed to reduce the

dimensionality of the data and identify potential outliers. Missing values were imputed using the Barycenter ("v2-mnar") method from the msImpute R package (v. 1.7.0). Differential expression analysis was conducted using the limma R package, applying empirical Bayes moderation to improve statistical power. Proteins were deemed significantly differentially expressed if they passed a false discovery rate (FDR) threshold of ≤ 5% after Benjamini–Hochberg (BH) correction. Data visualisation was conducted using the ggplot2 R package.

FLAG-IP and mass spectrometry of human PSKH1-3C-FLAG

HEK293T cells were cultured in Dulbecco's modified Eagle medium (Lonza) supplemented with 10% fetal bovine serum (ThermoFisher Scientific, catalog #10099141), penicillin (50 U/ml), and streptomycin (0.25 μg/ml) (Lonza) and maintained at 37°C in 5% CO_2 humidified atmosphere. PSKH1 was cloned into a pcDNA3 backbone vector and expressed in frame with a C-terminal, 3C protease-cleavable FLAG epitope tag. HEK293T cells were transfected with either PSKH1-3C-FLAG or pcDNA3 EGFP as a control using a 3:1 polyethylenimine (PEI [branched average *M*w ~25 000 Da; Sigma–Aldrich]) to DNA ratio (30: 10 μg, for a single 10 cm culture dish). For immunoprecipitation (IP) experiments (all performed in triplicate), proteins were harvested 48 h post transfection in a lysis buffer containing 50 mM Tris–HCl (pH 7.4), 150 mM NaCl, 0.1% (v/v) Triton X-100, 1 mM DTT, 1% (w/v) dodecyl-β-D-Maltoside (DDM), 1 mM ethylenediaminetetraacetic acid (EDTA), 1 mM ethylene glycol-bis(β-aminoethyl ether)-*N*,*N*,*N′*,*N′*-tetraacetic acid (EGTA) and 5% (v/v) glycerol and supplemented with a protease inhibitor cocktail tablet and a phosphatase inhibitor tablet (Roche). Lysates were briefly sonicated on ice, clarified by centrifugation at 20 817×*g* for 20 min at 4°C, and the resulting supernatants were incubated with anti-FLAG G1 Affinity Resin (GenScript) for 3 h with gentle agitation at 4°C. Affinity beads containing bound protein were collected and washed three times in 50 mM Tris–HCl (pH 7.4) and 150 mM NaCl and then equilibrated in storage buffer (50 mM Tris–HCl [pH 7.4], 100 mM NaCl, 1 mM DTT, 1% (w/v) DDM and 5% (v/v) glycerol). The purified proteins were then eluted from the suspended beads over a 3 h period with 3C protease (0.5 μg) at 4°C, with gentle agitation.

IP preparations were diluted to 180 μL in 100 mM ammonium bicarbonate pH 8.0 and reduced and alkylated with dithiothreitol and iodoacetamide, as previously described (15). Samples were subject to SP3- bead digestion as described by (16) with the adaptions of: using 100 mM ammonium bicarbonate pH 8.0, 0.5 μg of Trypsin gold (Promega) and post-digest bead washing in 1% (w/v) Rapigest (Waters). Once supernatants were combined, samples were acidified by addition of a final concentration of 0.5% TFA and incubated for 30 min each at 37°C with 600 rpm shaking and on ice. Samples were centrifuged at 13,000 *g* for 10 min at 4°C and cleared supernatant collected into fresh tubes prior to vacuum centrifugation. Dried peptides were solubilized in 20 μl of 3% (v/v) acetonitrile and 0.1% (v/v) TFA in water, sonicated for 10 minutes, and centrifuged at 13,000 ×*g* for 15 min at 4°C prior to reversed-phase HPLC separation using an Ultimate3000 nano system (Dionex) over a 60-minute gradient, as described by (15). Data were acquired using a Thermo QExactive mass spectrometer (Thermo Fisher Scientific), with higher-energy C-trap dissociation (HCD) fragmentation set at 30% normalized collision energy for 2+ to 4+ charge states. MS1 spectra were acquired in the Orbitrap (70K resolution at 200 *m/z*) over a *m/z* range of 300 to 2000, AGC target = $1e^6$, maximum injection time = 250 ms, with an intensity threshold for fragmentation of $1e^3$. MS2 spectra were acquired in the Orbitrap (17,500 resolution at 200 *m/z*), maximum injection time = 50 ms, AGC target = $1e^5$ with a 20 s dynamic exclusion window applied with a 10 ppm tolerance. Data was analysed using Proteome Discoverer 2.4 (ThermoFisher Scientific) in conjunction with MASCOT (17); searching the UniProt Human Reviewed database (updated weekly, accessed September 2024) with constant modifications = carbamidomethyl (C), variable modifications = oxidation (M), instrument type = electrospray ionization–Fourier-transform ion cyclotron resonance (ESI-FTICR), MS1 mass tolerance = 10 ppm, MS2 mass tolerance = 0.01 Da. Analysis was performed as a single study with label free quantification performed using the Minora feature detector node, calculating the area under the curve for *m/z* values, total protein abundance was determined using the HI3 method (18). Imputation of missing values was performed using low abundance resampling. Data were exported as an Excel file and imported into a custom R script to calculate fold change and t-test statistics for plotting.

Transient Expression and Immunoprecipitation

Full-length PSKH1 with a C-terminal HA-tag was expressed in HEK293T cells grown in DMEM media (ThermoFisher Scientific), supplemented with 8% (v/v) Fetal Calf Serum (FCS; Thermo Fisher Scientific) at 37 °C with 5% CO_2. The cells were transfected at 60% confluency using FuGene HD (Roche Applied Science) with 2 µg of pcDNA3.1(-) vector DNA. After 48 h, transfected cells were harvested by rinsing with ice-cold PBS, followed by rapid lysis in situ using lysis buffer (50 mM Tris-HCl pH 7.4, 1% (v/v) Triton X-100, 1 mM PMSF, 1 mM EDTA, 150 mM NaCl, 2 mM Sodium Vanadate, 10 mM NaF) containing a cOmplete protease inhibitor tablet (Roche). Insoluble debris were removed by centrifugation and supernatants were mixed with 100 µL of anti-HA agarose (50% v/v; Sigma) pre-equilibrated in lysis buffer, followed by successive washes in lysis buffer containing 1 mM NaCl, and finally resuspended in 50 mM HEPES, pH 7.4. Total protein content was quantified using the BCA Protein Assay (ThermoFisher Scientific), according to manufacturer's instructions.

In Vitro Kinase Assays

PSKH1 activity was determined by measuring the transfer of radiolabelled phosphate from [γ-^{32}P]-ATP to a synthetic peptide substrate (ADR1; LKKLTRRASFSGQ; synthesized by GenScript, New Jersey, USA). Briefly, purified recombinant GFP-PSKH1 (10 ng) or 10 µL of PSKH1 immobilized on anti-HA agarose beads (50% v/v) were incubated in assay buffer (50 mM HEPES, pH 7.4, 1 mM DTT) containing 200 µM ADR1 peptide, 200 µM [^{32}P]-γ-ATP (Perkin Elmer, MA, USA), 5 mM $MgCl_2$ (Sigma) in a 30 µL assay for 10 min at 30 °C. Unless outlined below, all assays were performed analogously. For assays with PSKH1 truncations, reactions were also supplemented with 100 µM $CaCl_2$, 1 µM recombinant Calmodulin (CaM; produced in-house) and/or 1 µM recombinant RCN3 or UNC119B (produced in-house). For the RCN1/RCN3/CALU assays, 1 mM $CaCl_2$ and 25 mM $MgCl_2$ were used. For CaM or RCN3 dose-dependence assays, a titration range of CaM or RCN3 (0, 1, 2, 5, 10, 20, 50, 100, 200, 500, 1000 nM) was used, with either 5 or 25 mM $MgCl_2$, respectively. For $CaCl_2$ dose-dependence assays, a titration range of $CaCl_2$ (0, 0.2, 0.5, 1, 2, 5, 10, 20, 50, 100, 1000 µM) was used, alongside 1 µM recombinant CaM or RCN3. For experiments in Figure 4C with EGTA, assays were supplemented with 1 mM EGTA. Reactions were terminated by spotting 15 µL of each reaction onto phosphocellulose paper (SVI-P; SVI, Melbourne, Australia) and washing extensively in 1% phosphoric acid (Sigma) and dried. Radioactivity was quantified by liquid scintillation counting.

Chemical crosslinking sample preparation

Recombinant full-length PSKH1 and either recombinant CaM, RCN3 or UNC119B (1:2 molar ratio) were initially incubated on ice in 50 mM HEPES (pH 7.5), 1 mM $CaCl_2$ (absent for UNC119B assay) for 30 min. Protein (1 mg/mL) was then mixed with SDA (NHS-Diazirine; 1 mg/mL; ThermoFisher Scientific #26167) and incubated in the dark for 30 min at room temperature to react the NHS-ester group. The diazarine group was then photo-activated (1 min pulse) using ultraviolet light irradiation (UVP CL-1000L UV cross-linker) at 365 nm. Samples were added to upturned lids excised from 1.5 mL microfuge tubes and placed on ice at a distance of 5 cm from the lamp and irradiated for 1 min. The reaction mixtures from the titration were combined and quenched with 100 mM Tris-HCl (pH 7.5), mixed with reducing sample buffer, heated at 100°C for 5 min, then resolved by reducing SDS-PAGE gel electrophoresis (Bio-Rad). SDS-PAGE gels were stained using SimplyBlue SafeStain (ThermoFisher Scientific) and crosslinked adducts excised for mass spectrometry analysis.

Chemical crosslinking sample preparation for mass spectrometry-based proteomics

Protein gel bands were excised (based on their migration relative to the MW marker) and destained using 50% acetonitrile for 30 min and 37°C. The gel band was then dehydrated by adding 100% acetonitrile (ACN) and incubating at room temperature for 10 min, before aspirating the ACN and further drying gel piece with the vacuum centrifuge (CentriVap, Labconco). Proteins were reduced by the addition of 1 mM dithiothreitol (DTT) in 50 mM ammonium bicarbonate for 30 min. Excess DTT was aspirated before the addition of 55 mM iodoacetamide to alkylate the sample for 60 min

at room temperature. The gel slice was then washed with 50% acetonitrile twice and 100% ACN once before drying to completion in the CentriVap vacuum centrifuge. Proteins were digested overnight with 500 ng trypsin in 50 mM ammonium bicarbonate at 37°C and extracted the following day using 60% acetonitrile/0.1% formic acid. The collected peptides were lyophilized to dryness using a CentriVap (Labconco), before reconstituting in 10 µL 0.1% formic acid/2% ACN ready for mass spectrometry analysis.

Chemical crosslinking mass spectrometry analysis

Reconstituted peptides were analyzed on Orbitrap Eclipse Tribrid mass spectrometer interfaced with Neo Vanquish liquid chromatography system. Samples were loaded onto a C18 fused silica column (inner diameter 75 µm, OD 360 µm × 15 cm length, 1.6 µm C18 beads) packed into an emitter tip (IonOpticks) using pressure-controlled loading with a maximum pressure of 1,500 bar using Easy nLC source and electro sprayed directly into the mass spectrometer. We first employed a linear gradient of 3-30% of solvent-B at 400 nL/min flow rate (solvent-B: 99.9% v/v ACN) for 100 min, followed by a gradient of 30-40% solvent-B for 20 min and 35-99% solvent-B for 5 min. The column was then maintained at 99% B for 10 min before being washed with 3% solvent-B for another 10 min comprising a total of 145 min run with a 120 min gradient in a data dependent (DDA) mode. MS1 spectra were acquired in the Orbitrap (R = 120k; normalised AGC target = standard; MaxIT = Auto; RF Lens = 30%; scan range = 380–1400; profile data). Dynamic exclusion was employed for 30 s excluding all charge states for a given precursor. Data dependent MS2 spectra were collected in the Orbitrap for precursors with charge states 3-8 (R = 50k; HCD collision energy mode = assisted; normalized HCD collision energies = 25%, 30%; scan range mode = normal; normalised AGC target = 200%; MaxIT = 150 ms). MGF files were searched against a fasta file containing the PSKH1 and interactor sequences using XiSearch software (19) (version 1.7.6.7) with the following settings: crosslinker = multiple, SDA and noncovalent; fixed modifications = Carbamidomethylation (C); variable modifications = oxidation (M), SDA-loop (KSTY) DELTAMASS:82.04186484, SDA-hydro (KSTY) DELTAMASS:100.052430; MS1 tolerance = 6.0ppm, MS2 tolerance = 20.0ppm; losses = H_2O, NH_3, CH_3SOH, CleavableCrossLinkerPeptide:MASS:82.04186484). FDR was performed with the in-built xiFDR set to 5%. Data were visualized using the XiView software (20).

Computational Modelling of PSKH1

The AlphaFold model of PSKH1 was obtained from the AlphaFold Protein Structure Database (EMBL-EBI) (21). Models of PSKH1 in complex with binding partners (Calmodulin, RCN3 and UNC119B) were generated using the AlphaFold3 server (22). Predicted Alignment Error (PAE) plots of the each PSKH1 complex are shown in Figure S4. Richardson (Ribbons) and surface diagrams were drawn using UCSF Chimera.

Western blot

SDS Laemmli lysis buffer (2×) was added to cells (or HA-pulldown), sonicated, boiled at 100 °C for 5 min, and then resolved on a 4-15% Tris-Glycine gel (Bio-Rad). After transfer to PVDF, membranes were blocked with 5% skim milk and then probed with primary antibodies (as per *Antibodies* below). The signals were revealed by enhanced chemiluminescence on a ChemiDoc Touch Imaging System (Bio-Rad) using an appropriate HRP-conjugated secondary antibody (as per *Antibodies* below).

Antibodies

Primary antibodies used in this study for immunoblotting were: rat anti-FLAG (WEHI clone 9H1; 1:1000); rat anti-human PSKH2 (WEHI clone 1B12; 1:1000); and rabbit anti-HA tag (Cell Signaling Technology; clone C29F4; 1:1000). Secondary antibodies used in this study were: horseradish peroxidase (HRP)-conjugated goat anti-rat IgG (Southern Biotech 3010-05); and HRP-conjugated goat anti-rabbit IgG (Cell Signaling Technology; clone 7074). All secondary antibodies were used at a dilution of 1:10000.

Rat anti-PSKH2 monoclonal antibody production

A synthetic peptide coupled to KLH and unique to PSKH2 (GPEAAAQAAQRIQVARFRAK; synthesized by Mimotopes, Australia), was used to immunize Wistar rats at the Walter and Eliza Hall Institute Monoclonal Antibody Facility to generate antibodies, as described before (23, 24). Splenocytes from immunized rats were fused with SP2/O mouse myeloid cells and the arising hybridoma lines single cell cloned. Hybridomas were then screened against the same synthetic peptide coupled to BSA relative to a BSA only control by ELISA. The specificity of clone 1B12 for human PSKH2 was subsequently validated via immunoblot analyses with cell lysates and recombinant full-length PSKH2 protein, as exemplified in Figure S2A.

Supplementary Information Figures

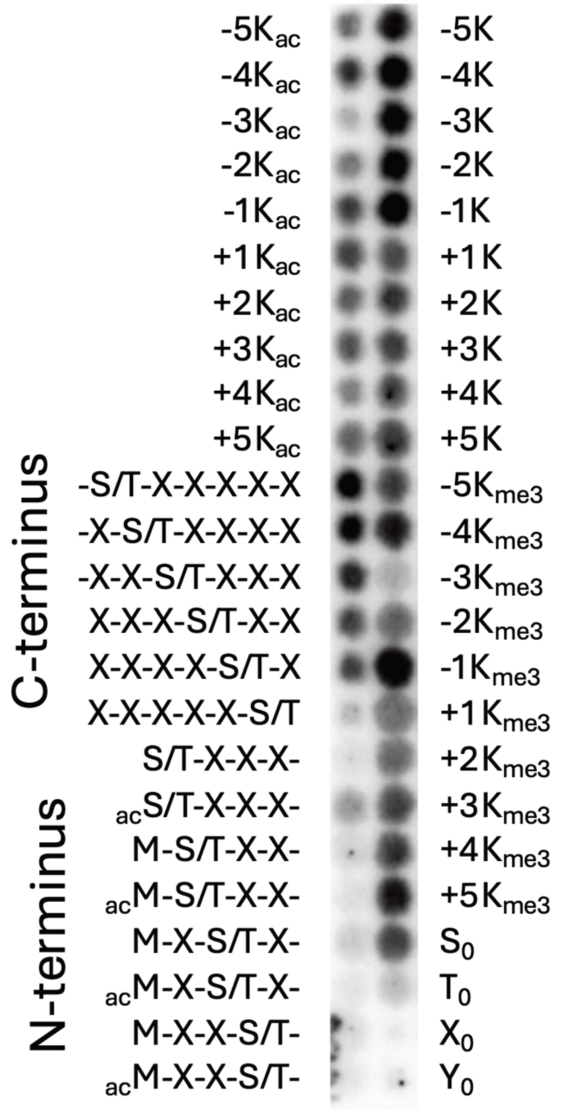

Fig. S1. PSKH1 prefers basic residues at the -3 position and favors C-terminal phosphoacceptors. Lysine (K) residues are well tolerated at all positions, although loss of charge by acetylation (Ac) or trimethylation (Me3) at the -3 position compromises recognition by PSKH1. S_0, G-A-X-X-X-X-X-S-X-X-X-X-A-G-K-K(LC-biotin); T_0, G-A-X-X-X-X-X-T-X-X-X-X-A-G-K-K(LC-biotin); X_0, G-A-X-X-X-X-X-X-X-X-X-X-A-G-K-K(LC-biotin); Y_0, G-A-X-X-X-X-X-Y-X-X-X-X-A-G-K-K(LC-biotin); where X = degenerate mixture of the 16 natural amino acids excluding cysteine, tyrosine, serine, and threonine. The shown panel is a subsection of the Positional scanning peptide array experiment shown in Figure 1.

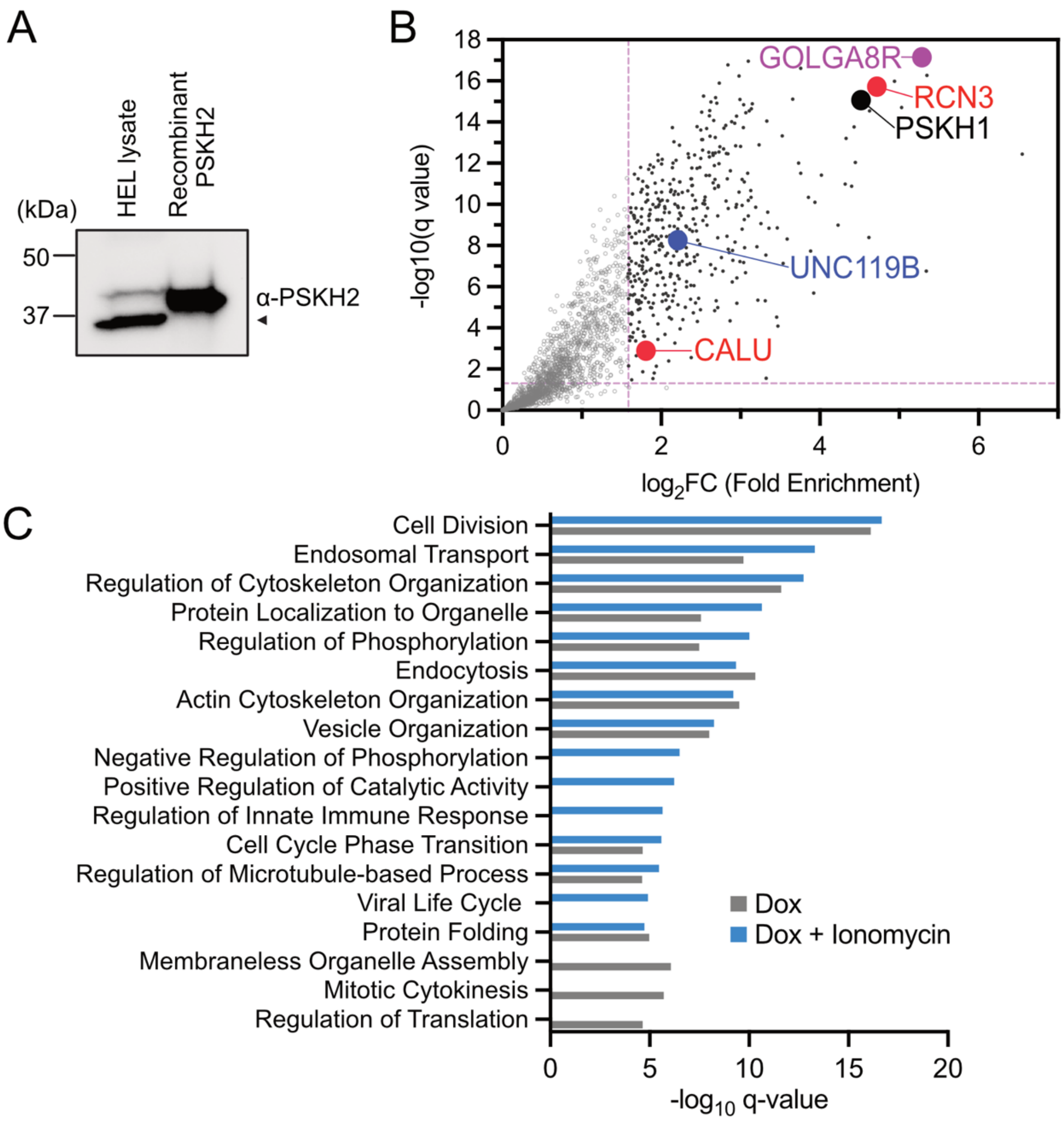

Fig. S2. Analysis of PSKH1 interactome. A While the PSKH2 pseudokinase is absent from the PSKH1 TurboID interactome, PSKH2 can be detected in the lysates of HEL cells used in the experiment. HEL cell lysates were probed with anti-PSKH2 (produced in house; clone 1B12; 1:1000 dilution), followed by HRP-conjugated goat anti-rat IgG (Southern Biotech 3010-05; 1:10000 dilution). Detection of PSKH2 in HEL cells is validated against recombinant PSKH2. Possible PSKH2 splice isoform is highlighted by an arrow at 37 kDa. **B** Volcano plot of TurboID proximity labeling experiment in HEL cells, following ionomycin treatment, with PSKH1 and proximal proteins of interest highlighted. PSKH1 is highlighted in black, UNC119B in blue, Calcium sensing interactors of the CREC family in red, secretory pathway interactors in purple. Pink dashed lines denote a 3-fold change and p-value <0.05. Data are representative of 6 independent experiments. **C** Gene ontology (GO) enrichment analysis of TurboID experiment in the absence (grey; from Fig. 2B) and presence of ionomycin treatment (blue; from Fig. S2B). The most enriched genes are largely unchanged with respect to gene ontology.

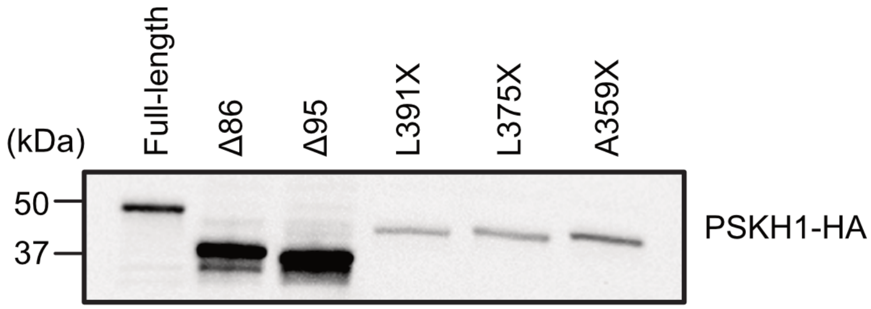

Fig. S3. Western blot analysis of wild-type HA-tagged PSKH1 and PSKH1 truncations affinity-purified from HEK293T cell lysates for in vitro kinase assays. HA-pulldowns were probed with anti-HA tag (Cell Signaling Technology; clone C29F4; 1:1000), followed by HRP-conjugated goat anti-rabbit IgG (Cell Signaling Technology; clone 7074; 1:10000).

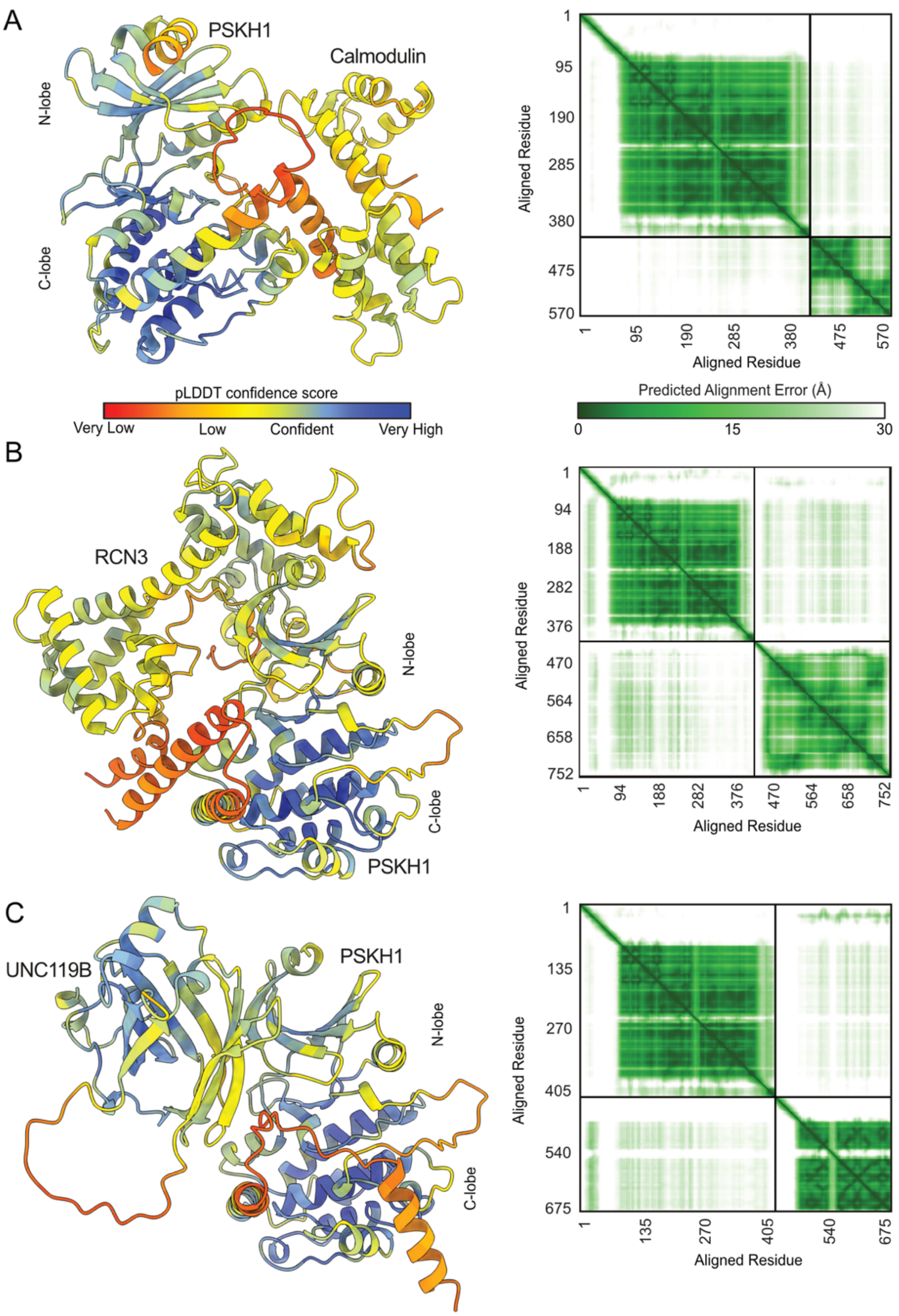

Fig. S4. Predicted Alignment Error (PAE) of AlphaFold models with PSKH1 in complex with **A)** Calmodulin, **B** RCN3 and **C** UNC119B. Left hand panels show the per-residue measure of local confidence score (pLDDT) mapped to each Richardson (ribbon) diagram. The rainbow legend for the pLDDT score applies to all models. Right side panels show the PAE plot for each model, where the green legend of the PAE in Å applies to all models. PSKH1 and each binding partner is annotated in each model.

Supplementary Information References

1. S. Maddirevula *et al.*, Large Scale Genomic Investigation of Pediatric Cholestasis Reveals a Novel Hepatorenal Ciliopathy Caused by PSKH1 Mutations. *Genet Med* 10.1016/j.gim.2024.101231, 101231 (2024).
2. J. M. Murphy *et al.*, The pseudokinase MLKL mediates necroptosis via a molecular switch mechanism. *Immunity* **39**, 443-453 (2013).
3. T. C. Branon *et al.*, Efficient proximity labeling in living cells and organisms with TurboID. *Nat Biotechnol* **36**, 880-887 (2018).
4. N. Suzuki *et al.*, Calcium-dependent structural changes in human reticulocalbin-1. *J Biochem* **155**, 281-293 (2014).
5. M. C. Tanzer *et al.*, Necroptosis signalling is tuned by phosphorylation of MLKL residues outside the pseudokinase domain activation loop. *Biochem J* **471**, 255-265 (2015).
6. D. P. Byrne *et al.*, Evolutionary and cellular analysis of the 'dark' pseudokinase PSKH2. *Biochem J* **480**, 141-160 (2023).
7. C. Fitzgibbon, Y. Meng, J. M. Murphy, Co-expression of recombinant RIPK3:MLKL complexes using the baculovirus-insect cell system. *Methods Enzymol* **667**, 183-227 (2022).
8. J. L. Johnson *et al.*, An atlas of substrate specificities for the human serine/threonine kinome. *Nature* **613**, 759-766 (2023).
9. Y. Meng *et al.*, Human RIPK3 C-lobe phosphorylation is essential for necroptotic signaling. *Cell Death Dis* **13**, 565 (2022).
10. V. Demichev, C. B. Messner, S. I. Vernardis, K. S. Lilley, M. Ralser, DIA-NN: neural networks and interference correction enable deep proteome coverage in high throughput. *Nat Methods* **17**, 41-44 (2020).
11. Y. Zhou *et al.*, Metascape provides a biologist-oriented resource for the analysis of systems-level datasets. *Nat Commun* **10**, 1523 (2019).
12. D. R. Croucher *et al.*, Bimolecular complementation affinity purification (BiCAP) reveals dimer-specific protein interactions for ERBB2 dimers. *Sci Signal* **9**, ra69 (2016).
13. J. F. Hastings *et al.*, Dissecting Multi-protein Signaling Complexes by Bimolecular Complementation Affinity Purification (BiCAP). *J Vis Exp* 10.3791/57109 (2018).
14. J. R. Wisniewski, A. Zougman, N. Nagaraj, M. Mann, Universal sample preparation method for proteome analysis. *Nat Methods* **6**, 359-362 (2009).
15. S. Ferries *et al.*, Evaluation of Parameters for Confident Phosphorylation Site Localization Using an Orbitrap Fusion Tribrid Mass Spectrometer. *Journal of Proteome Research* **16**, 3448-3459 (2017).
16. L. A. Daly *et al.*, Custom Workflow for the Confident Identification of Sulfotyrosine-Containing Peptides and Their Discrimination from Phosphopeptides. *Journal of Proteome Research* **22**, 3754-3772 (2023).
17. D. N. Perkins, D. J. Pappin, D. M. Creasy, J. S. Cottrell, Probability-based protein identification by searching sequence databases using mass spectrometry data. *Electrophoresis* **20**, 3551-3567 (1999).
18. J. C. Silva, M. V. Gorenstein, G.-Z. Li, J. P. C. Vissers, S. J. Geromanos, Absolute Quantification of Proteins by LCMSE: A Virtue of Parallel ms Acquisition *S. *Molecular & Cellular Proteomics* **5**, 144-156 (2006).
19. M. L. Mendes *et al.*, An integrated workflow for crosslinking mass spectrometry. *Molecular Systems Biology* **15**, e8994 (2019).
20. M. Graham, C. Combe, L. Kolbowski, J. Rappsilber (2019) xiView: A common platform for the downstream analysis of Crosslinking Mass Spectrometry data. (bioRxiv).
21. J. Jumper *et al.*, Highly accurate protein structure prediction with AlphaFold. *Nature* **596**, 583-589 (2021).
22. J. Abramson *et al.*, Accurate structure prediction of biomolecular interactions with AlphaFold 3. *Nature* **630**, 493-500 (2024).
23. A. L. Samson *et al.*, A toolbox for imaging RIPK1, RIPK3, and MLKL in mouse and human cells. *Cell Death Differ* 10.1038/s41418-021-00742-x (2021).

24. A. L. Samson *et al.*, MLKL trafficking and accumulation at the plasma membrane control the kinetics and threshold for necroptosis. *Nat Commun* **11**, 3151 (2020).
